# Discovery of an unknown diversity of *Leucinodes* species damaging Solanaceae fruits in sub-Saharan Africa and moving in trade (Insecta, Lepidoptera, Pyraloidea)

**DOI:** 10.3897/zookeys.472.8781

**Published:** 2015-01-19

**Authors:** Richard Mally, Anastasia Korycinska, David J. L. Agassiz, Jayne Hall, Jennifer Hodgetts, Matthias Nuss

**Affiliations:** 1 University Museum of Bergen, Realfagbygget, Allégaten 41, 5007 Bergen, Norway; 2 The Food and Environment Research Agency, Sand Hutton, York, YO41 1LZ, United Kingdom; 3 The Natural History Museum, London SW7 5BD, United Kingdom; 4 Senckenberg Natural History Collections Dresden, Königsbrücker Landstr. 159, 01109 Dresden, Germany

**Keywords:** *Leucinodes*, *Leucinodes orbonalis* complex, *Sceliodes*, Solanaceae, eggplant, pest species, DNA Barcoding, revision, identification key, Africa

## Abstract

The larvae of the Old World genera *Leucinodes* Guenée, 1854 and *Sceliodes* Guenée, 1854 are internal feeders in the fruits of Solanaceae, causing economic damage to cultivated plants like *Solanum melongena* and *Solanum aethiopicum*. In sub-Saharan Africa five nominal species of *Leucinodes* and one of *Sceliodes* occur. One of these species, the eggplant fruit and shoot borer *Leucinodes orbonalis* Guenée, 1854, is regarded as regularly intercepted from Africa and Asia in Europe, North and South America and is therefore a quarantine pest on these continents. We investigate the taxonomy of African *Leucinodes* and *Sceliodes* based on morphological characters in wing pattern, genitalia and larvae, as well as mitochondrial DNA, providing these data for identification of all life stages. The results suggest that both genera are congeneric, with *Sceliodes* **syn. n.** established as junior subjective synonym of *Leucinodes*. *Leucinodes orbonalis* is described from Asia and none of the samples investigated from Africa belong to this species. Instead, sub-Saharan Africa harbours a complex of eight endemic *Leucinodes* species. Among the former nominal species of *Leucinodes* (and *Sceliodes*) from Africa, only *Leucinodes laisalis* (Walker, 1859), **comb. n.** (*Sceliodes*) is confirmed, with *Leucinodes translucidalis* Gaede, 1917, **syn. n.** as a junior subjective synonym. The other African *Leucinodes* species were unknown to science and are described as new: *Leucinodes africensis* **sp. n.**, *Leucinodes ethiopica* **sp. n.**, *Leucinodes kenyensis* **sp. n.**, *Leucinodes malawiensis* **sp. n.**, *Leucinodes pseudorbonalis* **sp. n.**, *Leucinodes rimavallis* **sp. n.** and *Leucinodes ugandensis* **sp. n.** An identification key based on male genitalia is provided for the African *Leucinodes* species. Most imports of *Leucinodes* specimens from Africa into Europe refer to *Leucinodes africensis*, which has been frequently imported with fruits during the last 50 years. In contrast, *Leucinodes laisalis* has been much less frequently recorded, and *Leucinodes pseudorbonalis* as well as *Leucinodes rimavallis* only very recently in fruit imports from Uganda. Accordingly, interceptions of *Leucinodes* from Africa into other continents will need to be re-investigated for their species identity and will likely require, at least in parts, revisions of the quarantine regulations. The following African taxa are excluded from *Leucinodes*: *Hyperanalyta* Strand, 1918, **syn. rev.** as revised synonym of *Analyta* Lederer, 1863; *Analyta apicalis* (Hampson, 1896), **comb. n.** (*Leucinodes*); *Lygropia aureomarginalis* (Gaede, 1916), **comb. n.** (*Leucinodes*); *Syllepte hemichionalis* Mabille, 1900, **comb. rev.**, *Syllepte hemichionalis idalis* Viette, 1958, **comb. rev.** and *Syllepte vagans* (Tutt, 1890), **comb. n.** (*Aphytoceros*). *Deanolis iriocapna* (Meyrick, 1938), **comb. n.** from Indonesia is originally described and misplaced in *Sceliodes*, and *Leucinodes cordalis* (Doubleday, 1843), **comb. n.** (*Margaritia*) from New Zealand, *Leucinodes raondry* (Viette, 1981), **comb. n.** (*Daraba*) from Madagascar as well as *Leucinodes grisealis* (Kenrick, 1912), **comb. n.** (*Sceliodes*) from New Guinea are transferred from *Sceliodes* to *Leucinodes*. While *Leucinodes* is now revised from Africa, it still needs further revision in Asia.

## Introduction

*Leucinodes orbonalis* Guenée, 1854, the eggplant fruit and shoot borer, is a species of moth that was first described from specimens from India and Java (Guenée 1854). According to current knowledge, it is widely distributed in tropical and subtropical Asia (CABI 2012a) and sub-Saharan Africa (Walker 1859, Frempong 1979, CABI 2012a). The larvae are pests of Solanaceae, especially *Solanum melongena* L. (aubergine, eggplant or brinjal) fruits and stems where they feed internally. Their infestation can substantially reduce yields from aubergine cultivation, and yield losses of more than 65% have been recorded from Asia (EPPO 2008).

The larvae are commonly moved in international trade with plants and fruits, as their internal feeding and the resulting damage may not be visible during pre-export inspections. Thus, *Leucinodes orbonalis* is a quarantine pest of concern to a number of countries outside its native range. This includes the member countries of the European and Mediterranean Plant Protection Organisation (EPPO), where it was recommended as an addition to the alert list of pests in 2008 (EPPO 2008), and in 2012 transferred to the A1 list of pests recommended for statutory regulation (EPPO 2012, 2013). It is an A1 pest for several South American countries including Uruguay and Argentina (COSAVE 2006) and has repeatedly been intercepted in the USA (Whittle and Ferguson 1987, Solis 1999, 2006). In England and Wales, the Plant Health and Seeds Inspectorate (PHSI) regularly intercept *Leucinodes* Guenée, 1854 larvae inside aubergines from South Asia and West Africa (The Food and Environment Research Agency (Fera), unpublished data).

Due to the economic impact of *Leucinodes orbonalis*, the development of a genetically modified eggplant was initiated in 2005 in India by introducing a crystal protein gene (Cry1Ac) from the bacterium *Bacillus thuringiensis* Berliner, 1915 (*Bt*) into the plant (Sood 2012). The insecticidal effect of the crystal proteins makes eggplant less susceptible to infestations by larvae of *Leucinodes orbonalis*. After field trials and approval for commercial cultivation from government scientists in 2009, a moratorium on the commercialisation of *Bt* brinjal was imposed due to public concerns on food safety (Pandey 2010).

Until recently, all pyraloid larvae damaging Solanaceae fruits in Asia and Africa and intercepted from imports to Europe have been regarded as two species, *Leucinodes orbonalis* and *Sceliodes laisalis* (Walker, 1859). Hayden et al. (2013) and Gilligan and Passoa (2014) pointed out that *Leucinodes orbonalis* is restricted to Asia and that there are "three species in the *Leucinodes orbonalis* complex in Africa that are not conspecific with the Asian species. Our investigations reveal that an even largernumber of *Leucinodes* species are intercepted from Africa. This points to the question of the identity of the intercepted species as well as on further four species of *Leucinodes* known from Africa, *Leucinodes aureomarginalis* Gaede, 1916, *Leucinodes hemichionalis* (Mabille, 1900), *Leucinodes translucidalis* Gaede, 1917 and *Leucinodes vagans* (Tutt, 1890) (Nuss et al. 2003–2014). Besides *Leucinodes*, there is the similar genus *Sceliodes* Guenée, 1854, of which *Sceliodes cordalis* (Doubleday, 1843) and *Sceliodes laisalis* (Walker, 1859) are also pests on solanaceous crops in Australia (Davis 1964), New Zealand (Martin 2010) and Africa (CABI 2012b).

Here we taxonomically revise *Leucinodes* and *Sceliodes* and their species from continental sub-Saharan Africa, in order to delimit species and to allow their proper identification.

## Methods

Specimens were examined from the following institutions: private collection David J. L. Agassiz, Weston-super-Mare, Great Britain (coll. DJLA), Natural History Museum, London, Great Britain (BMNH), Invertebrate Reference Collection, Food and Environment Research Agency, Sand Hutton, England (Fera), International Centre of Insect Physiology and Ecology, Nairobi, Kenya (ICIPE), private collection Timm Karisch, Dessau, Germany (coll. Karisch), University of Oslo, Natural History Museum, Norway (NHMO), National Museum of Kenya, Nairobi, Kenya (NMK), National Plant Protection Organization, Wageningen, the Netherlands (NPPO), Muséum national d’Histoire naturelle, Paris, France (MNHN), Musée royal de l’Afrique centrale, Tervuren, Belgium (RMCA), Senckenberg Deutsches Entomologisches Institut, Müncheberg, Germany (SDEI), Senckenberg Museum für Tierkunde, Dresden, Germany (SMTD), National Museum of Natural History, Washington, D.C., U.S.A. (USNM), Museum für Naturkunde, Berlin, Germany (ZMHB), University of Copenhagen, Museum of Zoology, Denmark (ZMUC).

Larvae were sourced through quarantine interceptions of eggplant fruit from Africa and Asia at several ports of entry in England. Most larvae were studied alive and subsequently reared to adults in order to confirm the species identity. In large containments a few larvae were preserved by boiling them in water for 30–90 seconds, then transferred to 70% ethanol. After 3–5 days, the ethanol was replaced to limit dilution from body contents. Reared adults were killed soon after emergence, with cyanide, ammonia or by freezing at -20 °C for a minimum of 2 hours. Field-collected adults were attracted by artificial light and killed with cyanide. All adult specimens were subsequently pinned. Genital dissections of thoroughly dried specimens were performed according to Robinson (1976). Setal nomenclature follows Hinton (1946). The chaetotaxic descriptions and the setal map focus on those microscopic setae visible at 60 × magnification.

DNA was extracted using either the NucleoSpin tissue kit (Macherey-Nagel) according to the manufacturer’s instructions or using the Chelex-100 resin based method (Boonham et al. 2002). DNA extraction with the NucleoSpin tissue kit was performed following the procedure of Knölke et al. (2005), extracting DNA from the abdomen of adult specimens and subsequent dissection of the genitalia from the macerated abdomen. Extracted abdomina were stored in 70% ethanol until genitalia were dissected. For the Chelex-100 resin based method single legs or wings were removed from dried, pinned specimens using fine forceps and placed in individual 0.6 ml Eppendorf tubes. Briefly, 100 µl molecular-grade water was added to the tissue sample and ground using a micropestle. 100 µl of a 50% w/v chelex resin:water slurry was added, the sample heated to 95 °C for 5 minutes, centrifuged for 5 minutes and the supernatant transferred and stored at -20 °C prior to use.

PCR of the 5’ half of the cytochrome C oxidase subunit I (COI) gene, the so-called DNA Barcode (for Metazoa), was performed using primers HybLCO (Folmer et al. 1994, Wahlberg and Wheat 2008) and HybNancy (Wahlberg and Wheat 2008). For degraded material primer pairs HybLCO/K699 and Ron/HybNancy (Wahlberg and Wheat 2008) were used to amplify the COI Barcode in two fragments. PCR was performed in 25 µl reactions comprising 0.4u BIO-X-ACT Short DNA Polymerase (Bioline), 2.5 µl 10×OptiBuffer, 1.5 mM MgCl_2_, 200 nM each primer, 200 nM dNTP mix and 1–2 µl DNA (concentration as extracted). Cycling conditions were as follows: initial denaturation for 5min at 95 °C, 40 cycles with 1) 30 sec at 95 °C, 2) 30 sec at 48 °C, 3) 90 sec (HybLCO/HybNancy) or 60 sec (primers for degraded material) at 70 °C, final extension of 10 min at 70 °C. Alternatively primers LepF and LepR (Hajibabaei et al. 2006) were used in 25 µl PCR reactions using BIO-X-ACT Short 2× mix (Bioline), 400 nM each primer and 1–2 µl DNA (concentration as extracted). Cycling conditions were as follows: initial denaturation for 5 min at 94 °C, 35 cycles with 1) 30 sec at 94 °C, 2) 45 sec at 50 °C, 3) 1min at 72 °C, final extension of 10 min at 72 °C.

PCR products were visualised by separation in 1–1.5% agarose gels in 1 × TBE buffer (89 mM Tris-borate 2 mM EDTA) containing GelRed or ethidium bromide and visualised under UV light. PCR amplicons were cleaned up using ExoSAP-IT (Affymetrix) or QIAquick PCR purification kit (QIAGEN). Sequencing of both strands was performed by Eurofins MWG Operon (Germany) or in-house as follows. Sequencing PCRs were performed with BigDye Terminator v3.1 cycle sequencing kit (Applied Biosystems) using 5pm of the sequencing primer tails T7/T3 (Wahlberg and Wheat 2008) and 0.5–4 µl PCR product. Final clean-up was done via sodium acetate-ethanol precipitation. Sequencing was performed on a 3130 Genetic Analyzer (Applied Biosystems). PCRs, PCR clean-up and sequencing PCRs were performed on a Mastercycler ep gradient S (Eppendorf) or GeneAmp9700 (Applied Biosystems).

Obtained DNA sequences were proofread by eye and aligned using PhyDE 0.9971 (Müller et al. 2008) or MEGA version 4.1 (Tamura et al. 2007). Ambiguous Barcode nucleotides were coded according to the IUPAC Ambiguity Code (Cornish-Bowden 1985). Sequences were then checked for plausibility using BLAST with the *blastn* algorithm (Altschul et al. 1990; URL: http://blast.ncbi.nlm.nih.gov/Blast.cgi) as well as the BOLD Identification System (IDS, URL: http://www.boldsystems.org/index.php/IDS_OpenIdEngine). A 615 basepair fragment was analyzed with MEGA version 6 (Tamura et al. 2013), using the distance criterion and the Neighbor Joining (NJ) algorithm (Saitou and Nei 1987) with uncorrected p-distances (Srivathsan and Meier 2011). Statistical support was evaluated through 1,000 Bootstrap replicates. *Udea ferrugalis* (Hübner, 1796) was used to root the NJ tree.

We apply the morphospecies concept to our study. The DNA Barcode is used as additional source of information and as an identification tool for all developmental stages of African *Leucinodes* species. The Solanaceae species names mentioned in this study refer to their former context and do not necessarily correspond to the revised *Solanum* taxonomy by Knapp et al. (2013).

Label data of studied specimens were compiled in order to generate a distribution map. Geographical coordinates, if not given on the label, were obtained via Google Earth, Version 5.2.1.1588 and subsequently plotted on a map using DIVA-GIS, Version 7.2.3 (Hijmans et al. 2004).

## Data resources

The data underpinning the analyses reported in this paper are deposited in the Dryad Data Repository at doi: 10.5061/dryad.kk0n9.

## Results

### Leucinodes

Taxon classificationAnimaliaLepidopteraCrambidae

Guenée, 1854

Leucinodes Guenée, 1854. Type species: *Leucinodes orbonalis* Guenée, 1854
Sceliodes Guenée, 1854, **syn. n.** Type species: *Sceliodes mucidalis* Guenée, 1854
Daraba Walker, 1859 (synonymised by Hampson 1899). Type species: *Daraba idmonealis* Walker, 1859
Eretria Snellen, 1880 (synonymised by Hampson 1899; Shaffer et al. 1996: junior homonym of *Eretria* Robineau-Desvoidy, 1863). Type species: *Eretria obsistalis* Snellen, 1880
Leuctinodes South, 1897 (misspell.)

#### Diagnosis.

*Leucinodes* is characterized by a forewing pattern which includes a brown base, a white antemedian line which is distally brown edged; a median area that is ochreous or brown from the costa to the middle of wing, and red-brown from the middle of wing towards the dorsum; below the apex is a black-brown half moon-shaped patch (missing in *Leucinodes malawiensis* sp. n.), edged by a thin white postmedian line and a white line at the margin of wing. The hindwings are white with inconspicuous pattern elements. *Leucinodes* females with only one frenular bristle in the hindwing, female labial palps with elongated 3^rd^ meron, male genitalia with identical location of the fibula-sacculus process-complex (process lacking in *Leucinodes cordalis* (Doubleday, 1843), *Leucinodes laisalis* and *Leucinodes malawiensis*), female genitalia with fine granular sclerotization of ductus bursae (in most species), antrum with thickened mesocuticula, presence of lateral antrum pockets. Larvae are internal feeders in Solanaceae.

#### Redescription of adults.

**Head.** Frons conically bulged (Figs 11–12) to flat; labial palps porrect, brownish, 1^st^ meron on ventral side with forward-directed tuft, 3rd meron in males half as long as 2^nd^ meron, longer in females (Figs 11–12); maxillary palps minute or missing; haustellum well developed; eyes large, hemispherical; ocelli present; antennae ciliate, pedicel white to brown, flagellum light brown, cilia in males longer than basal antennal radius (except in *Leucinodes malawiensis*), in females shorter than antennal radius; vertex with whitish to brown scales at the collar and brown scales directed forward; chaetosemata absent.

**Thorax.** Dorsal side whitish to brown with whitish and dark brown scales mixed in; ventral side whitish; legs predominantly whitish, foreleg femur, tibia and epiphysis light to dark brown; tibial spurs 0, 2, 4 (fore-, mid-, hindleg) with outer spur 1/2 to 2/3 the length of inner spur.

**Wings.** Forewing white translucent, light brown or orange- to grey-brown, basal area light to dark brown, delimited by white and dark brown double line or in species with brown forewing ground colour by dark brown antemedian line; median area with pale to dark brown, sometimes very faint proximal discoidal stigma (absent in *Leucinodes malawiensis*); distal discoidal stigma pale to dark brown, reaching from costa to forewing centre; central dorsum with prominent orange to dark brown, broadly L-shaped or triangular spot connected or disconnected with distal discoidal stigma; postmedian line sinuate, faint and grey to grey-brown, white edged, with prominent subcostal bulge; apex brown to grey-brown coloured (absent in *Leucinodes malawiensis*), with slim strip of white at outer margin; margin dotted at veins, with large dots at apex and M_3_; fringe white to pale brown with dark interruption at apex and at M_3_ (absent in *Leucinodes malawiensis*). Hindwing in both sexes with one frenular bristle, ground colour whitish, middle of wing with one or two spots, often faint; postmedian line inconspicuous, bent towards spot at middle of wing; area below apex suffused by pale brown to grey; margin dotted at end of veins, with large dot at end of M_3_.

**Abdomen.** First segment whitish, remainder light-, orange- or dark brown to grey.

**Male genitalia.** Uncus neck constricted, head circular, with dorsal agglomeration of thick setae; narrow transtilla arms with central notch, in *Leucinodes ethiopica* with dorsad spike on each arm; vinculum saccus round to V-shaped, short to more or less elongated, with or without keeled tip; juxta oval, subulate, short rhombical or tongue-shaped, with semicircular base; valvae elongate triangular, tapering posteriorly, costa and posterioventral margin loosely covered with long setae; fibula (*fi* in Fig. 13) arising at central part of mesal wall of valva or near costa; sacculus (*sa* in Fig. 13) large, elongate oval, with distal sclerotized process (*sp* in Fig. 13), often in close association with fibula, process absent in *Leucinodes laisalis*, *Leucinodes malawiensis*; ventral margin of distal valvae with or without granulated area (*ga* in Fig. 13); phallus simple, with variously shaped sclerites at posterior apodeme, vesica with or without cornuti.

**Female genitalia.** Corpus bursae ovoid, membranous, without signa; ductus bursae membranous with delicate granulation, partly reaching into posterior corpus bursae; antrum short to long, slim to broader than ductus bursae, anterior part sometimes coiled, mesocuticula thickened (strongly stained with Chlorazol Black) and exocuticula (inner layer) partly sclerotized; ostium bursae with lateral membranous pockets, with or without oval sclerites; both apophyses pairs simple, apophyses anteriores normally stronger developed than posterior apophyses, with or without broadened central portion.

#### Immature stages.

**Larva.** Last instar larvae with pink dorsal integument, intersegmental areas cream or light pink, the ventral integument cream; strength of the colouration very variable, pink colour on majority of abdominal segments often interrupted laterally by a transverse cream line; head, prothoracic and anal shields mid brown with variable black markings; early instar larvae white or cream with brown pinacula and black head, prothoracic and anal shields. In older larvae the dorsal integument turns beige, then increasingly deeper pink as the moults progress, head and prothoracic shield brown; pinacula pale brown and prominent against the integument in all instars. The chaetotaxy of the thorax and first nine abdominal segments of the last instar is illustrated in Fig. 35. The relative size of pinacula and positions of setae are very variable intraspecifically. The head is mid to light brown, with variable black markings around ocelli and at genal angle; relative positions of cranial setae very unstable in the specimens examined. The prothoracic shield is light to dark brown with pale median sulcus and two variable dark markings: one along part of the posterior margin, strongest medially, and the other mediolaterally; usually additional darker spots bordering the median sulcus and extending laterally, spots very variable in extent and position; prothoracic L pinaculum crescent shaped with variable posterior extension, L setae anterior to the spiracle, usually vertically aligned; microscopic seta MV3 clearly visible in most specimens, can be almost as prominent as the V seta; MV3 setae share a mid-ventral pinaculum or are on separate pinacula; meso- and metathorax with clearly visible dorsal and subdorsal microscopic pinacula at 60 × magnification; three ventral microscopic setae less prominent, with MV3 usually being the least evident, these with or without small pinacula. Many larvae are asymmetrical in this feature, with a pinaculum on one side, and seta only on the other. On the abdomen, there is one SV seta on segment 1, three SV setae on a single pinaculum on segment 2; microscopic seta MV3 visible on both segments 1 and 2, not prominent; microscopic setae on segments 3–8 mostly not visible at 60 × magnification; prolegs with crochets in a mesopenellipse; anal shield often lighter brown than pinacula, usually darker pigmented in anterior half.

**Pupa.** (Figs 42–46) Yellow to pale brown, lightly sclerotized, developing adult clearly visible as development proceeds; two distinct, raised hood-like structures dorsal to spiracles on abdominal segments 2 and 3 (Figs 42, 44); four pairs of long hooked setae ventral to cremaster; cocoon stout leathery, made of silk, firmly attached to the substrate.

#### Remarks.

*Sceliodes* and *Leucinodes* have traditionally been distinguished by their forewing ground colour, which is predominantly orange-brown to greyish-brown in *Sceliodes* and white translucent in *Leucinodes*. The newly discovered *Leucinodes ethiopica* sp. n. is intermediate in this character whereas all other wing pattern elements are homologous among *Leucinodes* and *Sceliodes* species. Study of the genitalia showed that cornuti are present in *Sceliodes* species, but are absent in *Leucinodes*, including *Leucinodes ethiopica*. The female genitalia contain oval to semicircular sclerites in the lateral antrum pockets of *Leucinodes ethiopica*, African *Sceliodes* and *Sceliodes cordalis*, the type-species of *Sceliodes*, which is distributed in Australia and New Zealand. Thus, there is a continuous variation between *Leucinodes* and *Sceliodes* and we here synonymise *Sceliodes* syn. n. with *Leucinodes*. As *Leucinodes* and *Sceliodes* have been published on the same date and in the same work, we here give precedence to *Leucinodes* as it is the better known of the two names, acting as first reviser according to ICZN 24.2.2.

### Leucinodes orbonalis

Taxon classificationAnimaliaLepidopteraCrambidae

Guenée, 1854

Figs 1 , 11–12 , 13 , 23 , 31 , 35 , 36–37

Leucinodes orbonalis Guenée 1854: 223.

#### Type-localities.

Bangladesh, Sylhet (male syntype); Java (female syntype).

#### Material examined.

**Type-specimens.** Syntype ♂ [rectangular whitish label with red border, red letters] “Typicum | Specimen”, [rectangular whitish label with black border] “Ex. Musaeo | Ach.Guénée”, [rectangular beige label] “Paravicini Coll. | B.M. 1937-383.”, [rectangular brownish label] “Orbonalis | Gn. Silhet”, [rectangular pale yellow label] “Leucinodes Gn. | orbonalis Gn. | Type ♂ 756.3.”, [square white label in red letters, slide number and gender in black] “Pyralidae | Brit.Mus. | Slide No. | 4496♂” (BMNH); syntype ♀ [rectangular whitish label with red border, red letters] “Typicum | Specimen”, [rectangular whitish label with black border] “Ex. Musaeo | Ach.Guénée”, [rectangular white label] “Paravicini Coll. | B.M. 1937-383.”, [rectangular pale yellow label] “Leucinodes Gn. | orbonalis Gn. | Type ♀ 756.3.”, transparent capsule with abdomen and left hindwing (BMNH). – **Additional material. VIETNAM.** 1♂ Lao Cai Province, surrounding of Mt. Fan Si Pan, Nui Se, 1927m, 22°21.168'N 103°46.477'E, 20./21.x.2001, leg. S. Löffler, prep. RM503, DNA Barcode BC MTD 01185 (SMTD); **SINGAPORE.** 1♂ 1♀ leg. H.N. Ridley, BMNH Pyralidae slides No. 23092 & No. 23100 (BMNH); **THE NETHERLANDS (IMPORT).** 1♂ Amsterdam (Schiphol), import Thailand, 22.xi.2006, ex larva 26.xi.2006, ex pupa 6.xii.2006, leg. S. Roes, det. M v. d. Straten, prep. RM641; 1♀ Amsterdam (Schiphol), import Thailand, 8.ii.2005, ex larva, leg. R. Hulzinga, det. M v. d. Straten, prep. RM642 (NPPO); **GREAT BRITAIN (IMPORT).** see Suppl. material 2 (Fera material).

#### Diagnosis.

Wing pattern indistinguishable from those of *Leucinodes africensis* sp. n., *Leucinodes rimavallis* sp. n., *Leucinodes pseudorbonalis* sp. n., *Leucinodes kenyensis* sp. n. and “*Leucinodes* spp.”, but distinguished from *Leucinodes malawiensis* by the absence of the forewing basal transversal streak and the presence of the apical half moon-shaped patch, and from *Leucinodes laisalis*, *Leucinodes ethiopica* and *Leucinodes ugandensis* sp. n. by the predominantly white forewing ground colour. Frons usually more strongly bulged than in other *Leucinodes* species, but *Leucinodes pseudorbonalis* can be very similar in this feature. In male genitalia distinguishable by: dorsal margin of valval sacculus concave; apical sclerotized sacculus process elongated cone-shaped and crossing with the similar-sized fibula (as in *Leucinodes pseudorbonalis*); juxta slender, tapering (similar in *Leucinodes africensis* and *Leucinodes rimavallis*); saccus of vinculum short, less prominent. Female genitalia: antrum only slightly bulged, exocuticula without sclerotized strip.

#### Redescription of adults.

**Head.** As for the genus, with frons strongly bulged (Figs 11–12).

**Figures 1–10.**
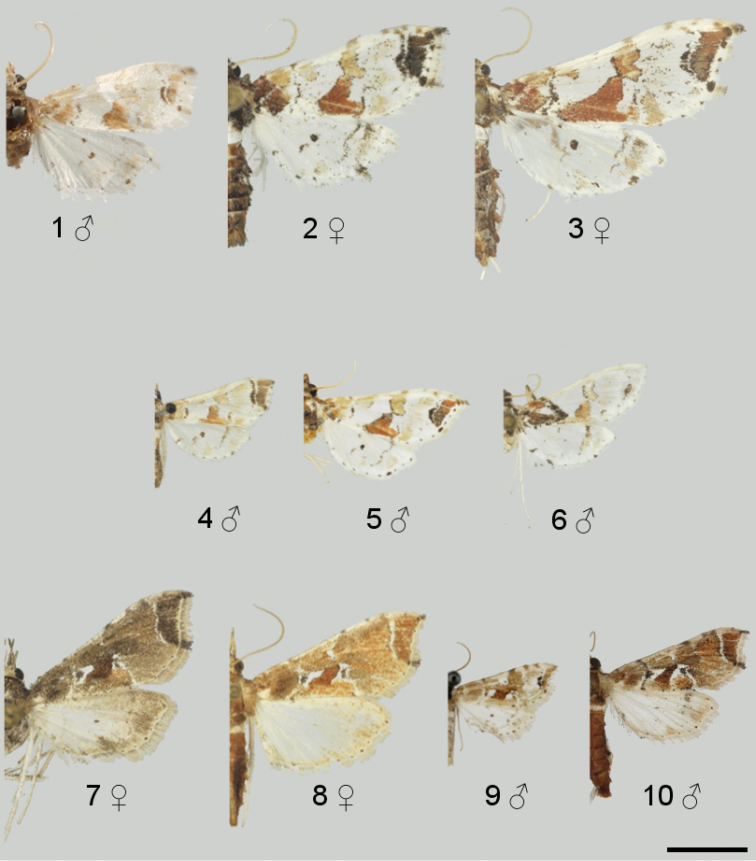
Adult specimens of *Leucinodes*. **1** *Leucinodes orbonalis*, syntype ♂ (Bangladesh) **2** *Leucinodes africensis* ♀ (Angola) **3** *Leucinodes rimavallis* ♀ (DR Congo: Kivu) **4** *Leucinodes pseudorbonalis* ♂ (Uganda) **5** *Leucinodes kenyensis*, holotype ♂ (Kenya) **6** *Leucinodes malawiensis*, holotype ♂ (Malawi) **7** *Leucinodes laisalis* ♀, greyish form (Tanzania) **8** *Leucinodes laisalis* ♀, brownish form (Tanzania) **9** *Leucinodes ethiopica*, holotype ♂ (Ethiopia) **10** *Leucinodes ugandensis*, holotype ♂ (Uganda). Scale bar represents 5 mm.

**Figures 11–12.**
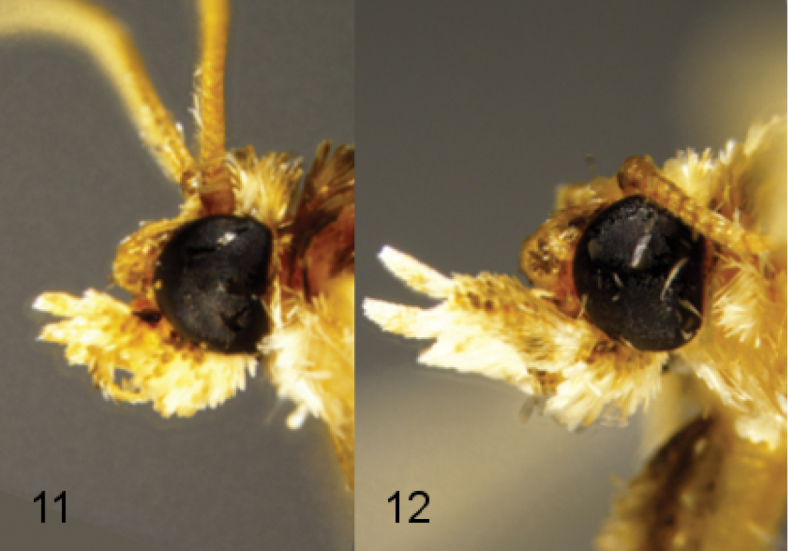
Head profiles of adult *Leucinodes orbonalis*. **11** male **12** female. Figures at same scale.

**Thorax.** As for the genus, with dorsal side brown.

**Wings.** Forewing length ♂ 8.5–10.5 m, ♀ 9.5–12.0 m; forewing ground colour white, basal area light- to dark brown, delimited by dark brown to grey antemedial line; median area with pale brown, faint proximal discoidal stigma; distal discoidal stigma pale brown, reaching from costa to forewing centre; central dorsum with prominent orange to dark brown L-shaped or triangular spot leading to forewing centre and often meeting with distal discoidal stigma; antemedial line sinuate, more or less distinct, but with prominent subcostal bulge; subapical half of termen with half moon-shaped brown to grey-grown spot; marginal line dotted; fringe and marginal line darkened at the tips of the half moon-shaped spot; hindwing ground colour white, internal area white, with discoidal spot, basicostally often with auxiliary spot; medial line sinuate, distal half approaching the discoidal spot, then turning towards dorsum; external area pale brown to gray; marginal line dotted.

**Abdomen.** First segment whitish, remainder brownish.

**Figures 13–22.**
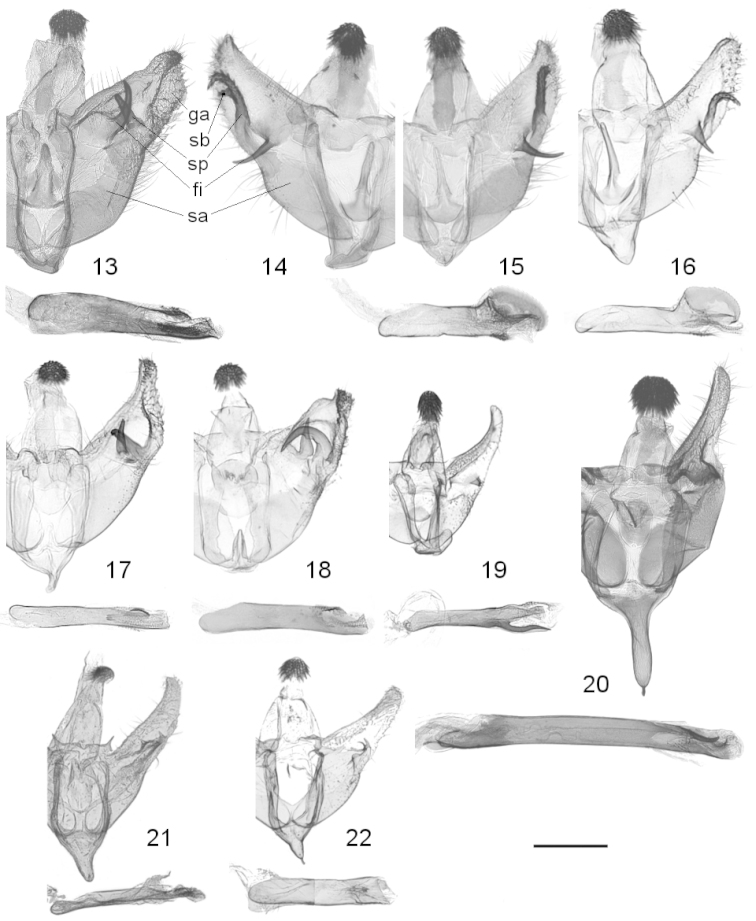
Male genitalia. **13** *Leucinodes orbonalis*, Vietnam (prep. RM503) **14** *Leucinodes africensis*, two-branched sacculus process, Côte d’Ivoire (prep. RM330, phallus omitted) **15** *Leucinodes africensis*, single-branched sacculus process, Ghana (import) (prep. RM501) **16** *Leucinodes rimavallis*, Kenya (prep. RM667) **17** *Leucinodes pseudorbonalis*, Uganda (prep. RM705) **18** *Leucinodes kenyensis*, Zimbabwe (prep. RM694) **19** *Leucinodes malawiensis*, Malawi (prep. RM683) **20** *Leucinodes laisalis*, South Africa (prep. RM504) **21** *Leucinodes ethiopica*, Ethiopia (BMNH Pyralidae slide 23138) **22** *Leucinodes ugandensis*, Somalia (BMNH Pyralidae slide 23140); phallus mirrored. Abbreviations: fi fibula, ga granulated area, sa sacculus, sb side branch of sacculus process, sp sacculus process. Scale bar represents 500 µm.

**Male genitalia.** As for the genus, apart from: juxta subulate, with short, broadly convex base, at 2/3 length slightly broadened; valvae broad, relatively short, nearly triangular; costa simple, slightly convex, subapically with a short concave portion; fibula hooked, its widened base emerging ventrad of costa base; sacculus ventrally convex, dorsally concave, at distal end with spike-shaped, strongly sclerotized process, oriented dorsad and crossing with fibula; distal ventral valva margin granulated (*ga* in Fig. 13), valva apex rounded, strongly granulated; distal 2/3 of ventral valva margin loosely covered with long thin setae; phallus simple, tapering posteriad, posterior apodeme dorsally elongate, ventrally with subapical, weakly serrated sclerite; ventral and dorsal portion of posterior apodeme separated by a slim, less strongly sclerotized region.

**Female genitalia.** As for the genus, apart from: anterior antrum with spoon-shaped posteriad indentation, flanked on either side by a sclerotized portion (*as* in Fig. 31); sternite 8 anterior edge arched (*ae* in Fig. 31), with a sclerite process leading in each of the lateral pockets; both apophysis pairs simple, slightly curved.

**Figures 23–27.**
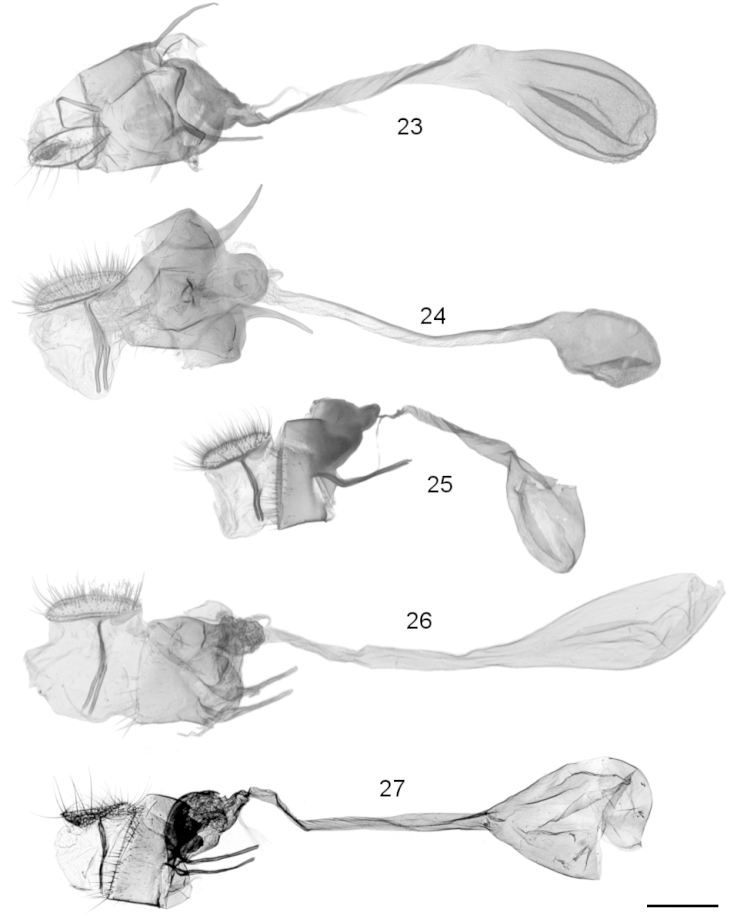
Female genitalia. **23** *Leucinodes orbonalis*, Thailand (import) (prep. RM642), ventral view **24** *Leucinodes africensis*, Ghana (prep. RM640), ventral view **25** *Leucinodes rimavallis*, Kenya (prep. RM666, SMTD Lep1592), lateral view **26** *Leucinodes pseudorbonalis*, Uganda (prep. RM706), lateral view **27** *Leucinodes kenyensis*, Kenya (prep. MN1134), lateral view. Scale bar represents 500 µm.

**Figures 28–34.**
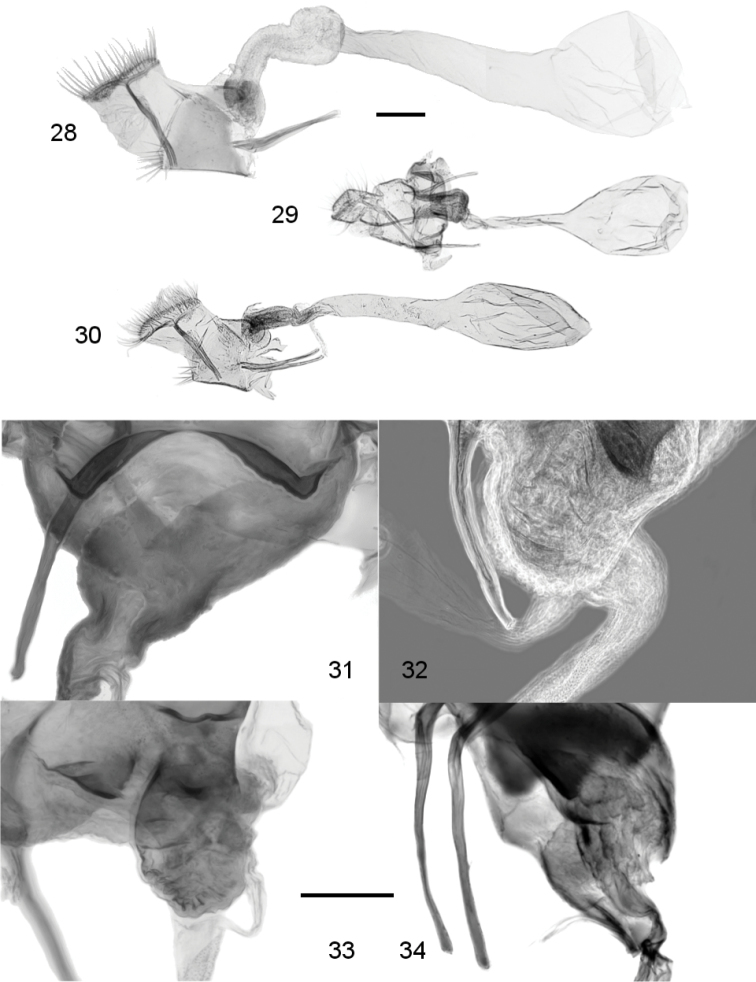
Female genitalia. **28** *Leucinodes laisalis*, Kenya (prep. RM308), lateral view **29** *Leucinodes ethiopica*, Ethiopia (BMNH Pyralidae slide 23139), ventral view **30** *Leucinodes ugandensis*, Somalia (BMNH Pyralidae slide No. 23137), lateral view **31** *Leucinodes orbonalis*, Thailand (import) (prep. RM642), ventral close-up of antrum region **32** *Leucinodes africensis*, Côte d’Ivoire (prep. RM743), dorsolateral close-up of antrum region (phase contrast filter) **33** *Leucinodes pseudorbonalis*, Uganda (prep. RM706), lateral close-up of antrum region **34** *Leucinodes kenyensis*, Kenya (prep. MN1134), lateral close-up of antrum region. Abbreviations: as antrum sclerotizations; Scale bar in **28–30** represents 500 µm and in **31–34** represents 200 µm.

#### Immature stages.

**Figure 35.**
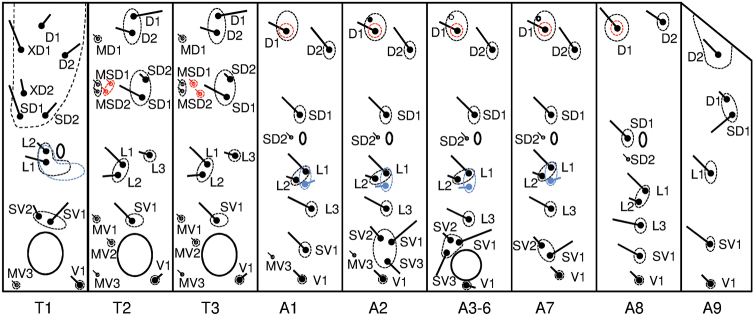
Chaetotaxy map of investigated *Leucinodes* larvae; blue elements illustrate variation found in *Leucinodes orbonalis* and *Leucinodes africensis*; red elements illustrate the differences found in *Leucinodes laisalis* compared to *Leucinodes orbonalis* and *Leucinodes africensis*.

**Larva.** MSD1 and MSD2 of meso- and metathorax usually on a shared pinaculum, earlier instars frequently have the MSD setae on separate pinacula on one or both segments; dorsal abdominal pinacula show apparent differentiation between West- and East-Asian populations: in live western specimens (e.g., from Pakistan), the abdominal D1 pinacula usually have an unpigmented cream coloured area near to their anteriomedian margin. This unpigmented area may be contiguous with the unmelanized cuticle surrounding the pinaculum or be surrounded by the melanized cuticle of the pinaculum (illustrated on A3–6 in Fig. 35); this cream white, unpigmented area may darken in preserved specimens, and in pre-pupae may be ringed in black. Eastern specimens (e.g., from Thailand) often have dark spots on at least some of the D1 pinacula (illustrated on A2 in Fig. 35); geographically intermediate populations (e.g., Sri Lanka) often show an intermediate form with black spots on occasional pinacula, and any unpigmented area is usually ringed with black pigmentation (illustrated on A7 in Fig. 35). East-Asian populations usually have mesally triordinal crochets, whereas the crochets of West-Asian populations are mesally biordinal. **Pupa.** Cremaster forms a variable shelf-like, sub-rectangular structure, much wider than long, usually with distinct distal corners and median notch; dorsal surface spinulose, with additional small but distinct spines which are variable in extent, form and number; cocoon of dark brown silk, may be white or beige when newly spun.

**Figures 36–41.**
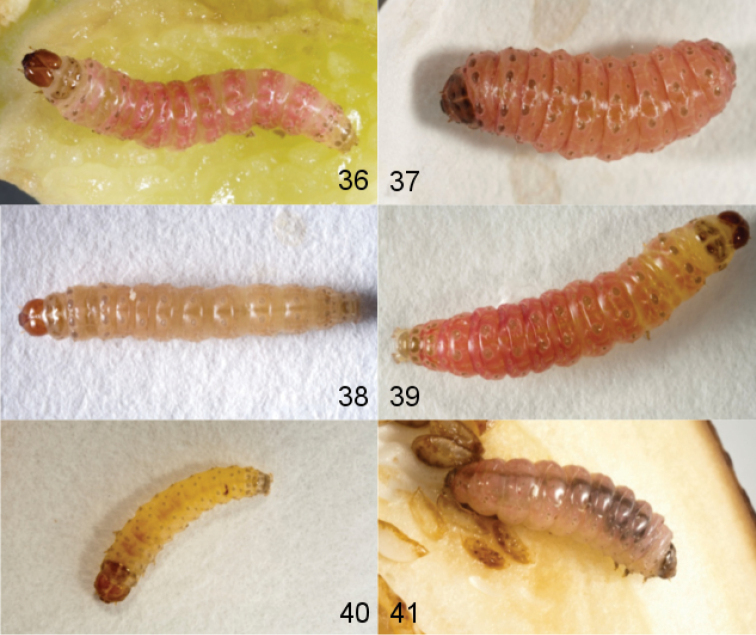
Larvae of *Leucinodes*. **36–37** *Leucinodes orbonalis* **36** mid instar **37** late instar **38–39** *Leucinodes africensis* **38** mid instar **39** late instar **40–41** *Leucinodes laisalis* **40** early instar **41** late instar.

#### Distribution.

India, Indonesia: Java (Guenée 1854), Sri Lanka (Walker 1859, Moore 1885), Myanmar (Burma), Andaman Islands (Pagenstecher 1900), Bangladesh, Brunei, Cambodia, China, Japan, Laos, Malaysia, Nepal, Pakistan, Philippines, Singapore, Taiwan, Thailand, Vietnam (CABI 2012a), Australia (Shaffer et al. 1996); imported to Great Britain, the Netherlands (pers. comm. M. van der Straten), Denmark (pers. comm. O. Karsholt) and the U.S.A. (Boateng et al. 2005, Solis 2006).

#### Food plants.

Solanaceae: *Solanum melongena* L., *Solanum aculeatissimum* Jacq., *Solanum aethiopicum* L., *Solanum erianthum* D. Don., *Solanum anguivi* Lam. (as *Solanum indicum* L.), *Solanum integrifolium* Poir., *Solanum lycopersicum* L., *Solanum macrocarpon* L., *Solanum mammosum* L., *Solanum nigrum* L., *Solanum torvum* Sw., *Solanum tuberosum* L., *Solanum viarum* Dunal, *Solanum xanthocarpum* Schrad., *Physalis minima* L., *Physalis peruviana* L., *Capsicum annuum* L. (van der Straten 2005; Hayden et al. 2013).

#### Remarks.

*Leucinodes orbonalis* has previously been reported in Europe from the Netherlands (van der Gaag et al. 2005) and Great Britain (Agassiz 1983; Higgott 2009) as well as from the following African countries due to misidentification: South Africa (Walker 1859; Pagenstecher 1900; Kemal and Koçak 2007); Kenya (Poulton 1916); Ghana (for example, Frempong and Buahin 1977, Frempong 1979, Duodu 1986, Horna et al. 2007); Lesotho, Zambia and Zimbabwe (Kopij 2005); Burundi, Cameroon, Congo, Democratic Republic of the Congo, Ethiopia, Malawi, Mozambique, Nigeria, Rwanda, Sao Tome and Principe, Sierra Leone, Somalia, Tanzania and Uganda (CABI 2012a). We have not found a single specimen from Africa belonging to this species and therefore postulate that *Leucinodes orbonalis* does not occur in Africa.

### Leucinodes africensis sp. n.

Taxon classificationAnimaliaLepidopteraCrambidae

http://zoobank.org/F8291E38-9C43-478D-8ED6-9C39EE507065

Figs 2 , 14–15 , 24 , 32 , 35 , 38–39 , 42–43

#### Type-locality.

West Africa, 11 June 1848, H. S. Le Marquand leg.

#### Material examined.

**Type-specimen.** Holotype ♂ [red-circled label] “Holo- | type”, “WEST AFRICA: | H.S. Le Marquand. | 11. xi. 48”, BM Pyralidae slide 23118 (BMNH). – **Additional material.** **GHANA.** 1♀ Kumasi, leg. J. D. G. Sanders, BMNH Pyralidae slide No. 23130 (BMNH); **LIBERIA.** 1♂ Kpaine, 7°10'N 9°07'W, 12.viii.1953, leg. Dr W. Peters, BMNH Pyralidae slide No. 23148 (BMNH); **CÔTE D’IVOIRE.** 1♂ Abidjan, 19.xi.1952, leg. L. Sheljuzhko, prep. RM330 (ZSM); 1♂ Bouaké, Inepa, 14.–15.vi.1983, col. Stam, prep. RM693 (RMCA); 1♀ Bingerville, 11.vi.1961, leg. J. Decelle, prep. RM704 (RMCA]; 1♀ Mont Nimba, Xealé, 6.ii.1959, leg. M. Condamin & R. Roy, prep. RM743 (MNHN); **NIGERIA.** 1♀ Lagos, 31.viii.1987, leg. Boorman, BMNH Pyralidae slide No. 23127 (BMNH); 1 ex. Oyo, Ibadan, International Institute of Tropical Agriculture, 7.501N 3.906E, 240m, 15.iii.2006, leg. S.E. Miller & T.M. Kuklenski, DNA Barcode USNM ENT 196725 (USNM); **GABON.** 1♂ Ntoum, xii.1986, leg. A. Pauly, prep. RM685 (RMCA); **DR CONGO.** 1♂ Sankuru, Dimbelenge, i.–ii. 1957, leg. M. Fontaine, prep. RM697 (RMCA); 1♀ Elisabethville, 20.ii.1934, leg. Ch. Seydel, prep. RM696 (RMCA); **ANGOLA.** 1♂ 3♀ prov. Uíge, Negage, market, 7°45'39.4"S 15°16'00.6"E, 1213 m, 21.iii.2013, fruits of *Solanum aethiopicum*, e.l. 19., 20., 21.iv.2013, leg. M. Nuss, 1♂ prep. RM643, DNA vouchers SMTD Lep1562 & Lep1563 (SMTD); 2♀ same data, but 30.i.2014, fruits of *Solanum aethiopicum*, e.l. 16.ii.2014, leg. M. Nuss (SMTD); **WEST AFRICA.** 1♂ ii.–xi.43, leg. H. S. Le Marquand, BMNH Pyralidae slide No. 23118 (phallus lost) (BMNH); **TANZANIA.** 2♀ Oldeani, 22.x.1961 & 9.xii.1961, leg. J. Killand, preps RM334 & RM634 (ZSM); **THE NETHERLANDS (IMPORT).** 1♂ Schiphol (Amsterdam), import Ghana, 18.ix.2009, ex larva 22.ix.2009, ex pupa 1.x.2009, leg. P. Dekker, det. M v. d. Straten, prep. RM501, DNA voucher SMTD Lep946, DNA Barcode BC MTD 01816 (NPPO); 1♀ Schiphol (Amsterdam), import Ghana, 18.ix.2009, ex larva 23.ix.2009, ex pupa 1.x.2009, leg. P. Dekker, det. M. v. d. Straten, prep. RM640 (NPPO); **GREAT BRITAIN (IMPORT).** 1 ♂ London Airport, import Zimbabwe (Rhodesia), 1965; 1♂ 1♀ import Nigeria, ex tomatoes, London Airport xi.1965 (FERA); for additional FERA material see Suppl. material 2.

#### Diagnosis.

The frons is less strongly bulged than in *Leucinodes orbonalis*. In wing pattern this species is indistinguishable from those of *Leucinodes orbonalis*, *Leucinodes rimavallis*, *Leucinodes pseudorbonalis*, *Leucinodes kenyensis* and “*Leucinodes* spp.”, but distinguished from *Leucinodes malawiensis* by the absence of the forewing basal transversal streak and the presence of the apical half moon-shaped patch, and from *Leucinodes laisalis*, *Leucinodes ethiopica* and *Leucinodes ugandensis* by the predominantly white forewing ground colour. In male genitalia it is distinguished by: the long ventrad fibula (as in *Leucinodes rimavallis*, short and triangular in *Leucinodes malawiensis*, broad and stout in *Leucinodes laisalis*); the elongate, straight or hook-shaped, sometimes branching distal sacculus process projecting towards the valva apex (similar in *Leucinodes rimavallis*); the apically thin, subulate juxta (similar in *Leucinodes rimavallis*); the prominent oval saw blade-shaped sclerotization of the posterior phallus apodeme (as in *Leucinodes rimavallis*); it is distinguished from *Leucinodes rimavallis* by the longer, more curved fibula with a slender base, the elongate distal sacculus process, which spans more than half the distance fibula base–valva apex, is straight or hook-shaped and sometimes exhibits a side branch, and the pointed valva apex (rounded in *Leucinodes rimavallis*). Female genitalia resemble those of *Leucinodes pseudorbonalis* in having a swollen antrum, but they lack the posterior constriction of the ostium bursae.

#### Description of adults.

**Head.** As for the genus, with frons moderately bulged, base of each meron of labial palps with white scales.

**Thorax.** As for the genus, with dorsal side brown.

**Wings.** Forewing length ♂ 7.5–10.5 m, ♀ 7.0–11.5 m; wing pattern as in *Leucinodes orbonalis*.

**Abdomen.** First segment whitish, remainder brown to grey.

**Male genitalia.** As for the genus, apart from: juxta base broad, semicircular, apical 2/3 of juxta thin, subulate; valvae broad, forming an oblong triangle; sacculus process porrect towards valva apex or apically bent, apex acanthaceous, sometimes with a similarly acanthaceous subapical side branch (*sb* in Fig. 14); ventrad fibula thin, spine-like, curved, crossing distal sacculus anterior to the sacculus process; valva apex pointed; posteriodorsal phallus apodeme with prominent oval saw blade-like sclerite, posterioventral apodeme with posteriodorsad oriented tapering process.

**Female genitalia.** As for the genus, apart from: colliculum-antrum complex in sagittal plane of sigmoid shape; dorsal surface of antrum exocuticle with longitudinal sclerotized strip running from sternite 8, bearing transverse ridges (Fig. 32); sternite 8 with anteriomedian recess, anteriolateral edges slightly dentate; apophyses anteriores with broadened central portion.

#### Immature stages.

**Larva.** Final-instar larvae of *Leucinodes africensis* and *Leucinodes orbonalis* cannot be definitively separated. In final instars of live specimens of *Leucinodes africensis*, the majority of the abdominal D1 pinacula have a dark pigmented spot on the anteriomedian margin (illustrated on A2 in Fig. 35), although in the occasional pinaculum this is replaced by an unpigmented area, which can be contiguous with the unmelanized integument surrounding the pinaculum or separated from it by the melanized cuticle of the pinaculum; crochets are mesally triordinal, as in the East-Asian populations of *Leucinodes orbonalis*. **Pupa.** length ca. 8.5 m; no consistent features separate the pupae of *Leucinodes orbonalis* and *Leucinodes africensis*.

**Figures 42–46.**
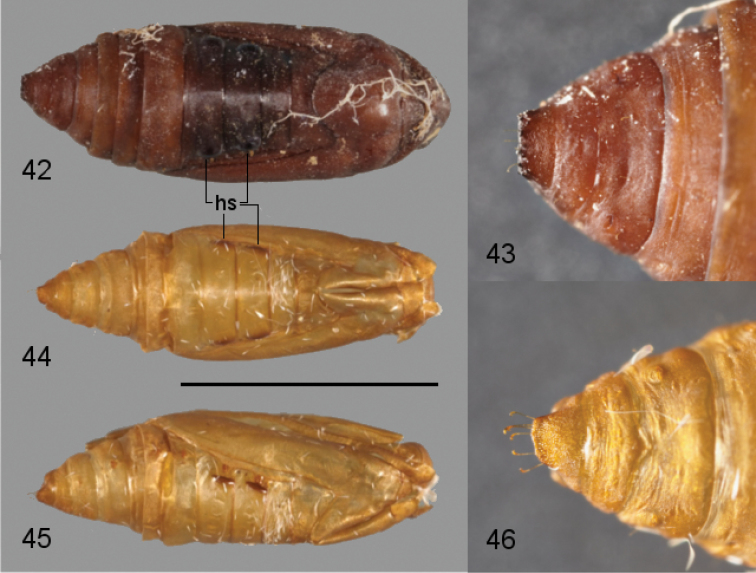
Pupae of *Leucinodes*. **42–43** *Leucinodes africensis* **42** dorsal view **43** close-up of cremaster **44–46** *Leucinodes laisalis* **44** dorsal view **45** lateral view **46** close-up of cremaster. Abbreviations: hs hood-like structures dorsal to spiracles on abdominal segments 2 and 3. Scale bar refers to **42, 44** and **45** and represents 5 mm.

#### Etymology.

Latinized *africensis*, derived from the continent of Africa from where the type material originates and referring to the widespread distribution of this species on the African continent.

#### Distribution.

Known from West Africa (Côte d’Ivoire, Ghana, Liberia, Nigeria), Angola, DR Congo, Gabon, and Tanzania; intercepted with plant imports from Ghana and Zimbabwe to Great Britain and the Netherlands. At least in the southern DR Congo (Lubumbashi) *Leucinodes rimavallis* occurs sympatrically with *Leucinodes africensis*.

#### Foodplants.

Solanaceae: *Solanum aethiopicum* L. (Angola, leg. Nuss 2013), *Solanum lycopersicon* L., *Solanum melongena* L.

#### Remarks.

This species is very similar to *Leucinodes rimavallis*, but both COI Barcoding data and constant morphological differences in genitalia separate the two species.

### Leucinodes rimavallis sp. n.

Taxon classificationAnimaliaLepidopteraCrambidae

http://zoobank.org/F7EFC84B-D824-420F-8D0D-F5D0FF026CE2

Figs 3 , 16 , 25

#### Type-lacality.

Kenya, Mt Elgon, 01°07'06"N, 34°41'30"E, February 1952, T. H. E. Jackson leg.

#### Material examined.

**Type-specimen.** Holotype ♂ [red-circled label] “Holo- | type”, “Mt Elgon | Kenya | Feb. 1952 | T.H.E. Jackson”, “Pres. by | Coryndon Mus. | B.M. 1961-696.”, B.M. Pyralidae Genitalia slide No. 23119 (BMNH). – **Additional material.** **RWANDA.** 1♂ Gisenyi (Kisenyi), 30.iv.1957, leg. M. Fontaine, prep. RM698 (RMCA); **BURUNDI.** 2♂ Kitega, 30.iv.1968 & 31.v.1969, leg. M. Fontaine, preps RM702 & RM703 (RMCA); **KENYA.** 1♂ Central Province, Castle Forest Lodge (S slope of Mt Kenya), 2050m, 0°21'15"S 35°18'12"E, 20.xi.2009, prep. DJLA1337 (coll. DJLA); 1♂ Central Province, Gatamayu Forest, 0°58.45'S 36°41.83'E, 21.vii.2001, leg. R.S. Copeland, DNA voucher SMTD Lep1593, prep. RM667 (RMCA); 1♂ Central Province, Gatamayu Forest, 0°58.45'S 36°41.83'E, 2284m, 17.viii.2002, leg. R. Copeland, prep. RM684 (RMCA); 1♀ Coast Province, Buda Forest, 4°27.79'S 39°24.20'E, 25.iv.2002, ex fruits *Withania somnifera*, leg. R.S. Copeland, DNA voucher MTD Lep1592, prep. RM666 (RMCA); 1♀ Fort-Hall [Murang’a], 1330 m, i.1912, leg. Alluaud & Jeannel, prep. RM742 (MNHN); 1♀ Taveta, 750 m, iii.1912, leg. Alluaud & Jeannel, prep. RM744 (MNHN); 1♀ Rurunga, 1550 m, i.1912, leg. Alluaud & Jeannel, prep. RM745 (MNHN); 1♂ Western Province, Kericho, 2050m, 0°21'15"S 35°18'12"E, 31.viii.1999, prep. DJLA1317 (coll. DJLA); 1♂ Mt. Elgon, ii.1952, leg. T.H.E. Jackson, BMNH Pyralidae slide No. 23119 (BMNH); 1♀ Mt. Elgon, i.1959, leg. T.H.E. Jackson, BMNH Pyralidae slide No. 23129 (BMNH); **DR CONGO.** 1♂ Ituri, Nioka, 5.ix.1953, leg. J. Hecq, prep. RM692 (RMCA); 1♂ Lubumbashi (Elisabethville), 13.x.1938, leg. Ch. Seydel, prep. RM695 (RMCA); 1♂ N. Kivu Lake, Rwankwi, iv.1948, leg. J. V. Leroy, prep. RM700 (RMCA); **SOUTH AFRICA.** 1♀ Natal, prep. RM735 (MNHN); **THE NETHERLANDS (IMPORT).** 1♂ Barendrecht, import Uganda, 26.ii.2014, leg. Sluijs, on *Solanum melongena*, prep. RM756 (NPPO); 1♂ Rijnsburg, import Uganda, 12.ii.2014, leg. J. de Zeeuw, on *Solanum melongena*, prep. RM757 (NPPO).

#### Diagnosis.

Frons is moderately bulged; in wing pattern this species is indistinguishable from *Leucinodes orbonalis*, *Leucinodes africensis*, *Leucinodes pseudorbonalis*, *Leucinodes kenyensis* and “*Leucinodes* spp.”, but distinguished from *Leucinodes malawiensis* by the absence of the forewing basal transversal streak and the presence of the apical half moon-shaped patch, and from *Leucinodes laisalis*, *Leucinodes ethiopica* and *Leucinodes ugandensis* by the predominantly white forewing ground colour. In male genitalia it is distinguished by: the long ventrad fibula (as in *Leucinodes africensis*, short and triangular in *Leucinodes malawiensis*, broad and stout in *Leucinodes laisalis*); the granulate, hook-shaped distal sacculus process (similar in *Leucinodes africensis*, process smooth in *Leucinodes kenyensis*); the apically thin, subulate juxta (as in *Leucinodes africensis*, broad subulate in *Leucinodes orbonalis*); the prominent oval saw blade-shaped sclerotization of the posterior phallus apodeme (as in *Leucinodes africensis*); distinguished from *Leucinodes africensis* by the shorter, straight to slightly curved fibula with a broader base, the shorter, always hook-shaped distal sacculus process, and the rounded valva apex (pointed in *Leucinodes africensis*). Female genitalia have slender apophyses anteriores (as in *Leucinodes orbonalis*), the central antrum tube with a short, strongly sclerotized section (long strip in *Leucinodes africensis* and *Leucinodes pseudorbonalis*), and the anteriolateral edges of sternite 8 with triangular processes extending into the lateral antrum pockets (as in *Leucinodes kenyensis*).

#### Description of adults.

**Head.** As for the genus, with frons moderately bulged.

**Thorax.** As for the genus, with dorsal side brown, tegula scales whitish-brown.

**Wings.** Forewing length ♂ 8.5–12.0 mm, ♀ 7.0–14.0 mm; wing pattern as in *Leucinodes orbonalis*.

**Abdomen.** First segment whitish, remainder light to dark brown.

**Male genitalia.** As in *Leucinodes africensis*, but with the fibula short, more triangular and robust, straight or slightly curved; distal sacculus process short and always bent apically; valva apex rounded or stout.

**Female genitalia.** As for the genus, apart from: anterior antrum with short sclerotized section, central posterior antrum with diffuse weak sclerotization; sternite 8 on each side with anteriad triangular process extending into the lateral antrum pockets.

#### Etymology.

From latin *rima* for ‘rift’ and *vallis* for ‘valley’, referring to the African Rift Valley, the main distributional area of this species (as far as known).

#### Distribution.

Burundi, Eastern and Southern Democratic Republic of the Congo, Kenya, Rwanda, South Africa, Uganda (import).

#### Foodplants.

Solanaceae: *Solanum melongena* L., *Withania somnifera* (L.) Dunal.

### Leucinodes pseudorbonalis sp. n.

Taxon classificationAnimaliaLepidopteraCrambidae

http://zoobank.org/5B6C0A50-209B-407D-A689-4C8716D9EA18

Figs 4 , 17 , 26 , 33

#### Type-locality.

Angola, Huambo Province, Luimbale, Mt Moco, 1800-1900 m, 12°28'S, 15°10'S, 18 March 1934, K. Jordan leg.

#### Material examined.

**Type-specimen.** Holotype ♂ [red-circled label] “Holo- | type”, “Mt. Moco, | Luimbale, | 1800 - 1900m., | 18 March 1934.”, “Angola | (Dr K. Jordan)”, “Rothschild | Bequest | 1939-1.”, BM Pyralidae slide 23135 (BMNH). – **Additional material.** **SENEGAL.** 1♂ Dakar, 01.viii.1952, leg. A. Villiers (BMNH); **UGANDA.** 1♂ Masindi, 29.xii.1897, leg. Ansorge, BMNH Pyralidae slide No. 23125 (BMNH); 1♂ Labonga, Unyoro, 13.xii.1897, leg. Ansorge, BMBH Pyralidae slide No. 23126 (BMNH); 1♂ Nabagulo Forest, 15 m from Kampala, 25.x.–06.xi.1921, leg. W. Feather, BMNH Pyralidae slide No. 23145 (BMNH); 1♂, Ruwenzori Range, Ibanda, 4,700ft, 4.–12.ix.1952, leg. D.S. Fletcher, BMNH Pyralidae slide No. 23146 (BMNH); 1♂ Masindi, 30.x.1897, leg. Ansorge, prep. RM707 (BMNH); 2♂ Kampala, 1897, leg. Dr. Ansorge, BMNH Pyralidae slide No. 23149, prep. RM705 (BMNH); 1♀ same data, prep. RM706 (BMNH); **THE NETHERLANDS (IMPORT):** 1♀ Barendrecht, import Uganda, 26.ii.2014, leg. Sluijs, on *Solanum melongena*, prep. RM758 (NPPO); **GREAT BRITAIN (IMPORT).** see Suppl. material 2.

#### Diagnosis.

Frons is moderately to strongly bulged; Wing pattern indistinguishable from those of *Leucinodes orbonalis*, *Leucinodes africensis*, *Leucinodes rimavallis*, *Leucinodes kenyensis* and “*Leucinodes* spp.”, but distinguished from *Leucinodes malawiensis* by the absence of the forewing basal transversal streak and the presence of the apical half moon-shaped patch, and from *Leucinodes laisalis*, *Leucinodes ethiopica* and *Leucinodes ugandensis* by the predominantly white forewing ground colour. In male genitalia the prominent dorsad fibula and the fibula-like sacculus process are roughly of same size and run parallel or cross each other (as in *Leucinodes orbonalis*, fibula and fibula-like sacculus process very small in *Leucinodes ugandensis*). Very similar to male genitalia of *Leucinodes orbonalis*, but valva tips slimmer and more acute, juxta with larger hemicircular base, elongated saccus tip and more prominent oval sclerite at posterior phallus apodeme. In female genitalia discriminated by the globular, posteriorly somewhat constricted antrum with a longitudinal, sclerotized exocuticular strip bearing transversal ridges (as in *Leucinodes africensis*).

#### Description of adults.

**Head.** As for the genus, with frons moderately to strongly bulged.

**Thorax.** As for the genus, with dorsal side whitish.

**Wings.** Forewing length ♂ 7.0–8.5 mm, ♀9.0–11.0 mm; wing pattern as in *Leucinodes orbonalis*.

**Abdomen.** First segment whitish, remainder orange-brown.

**Male genitalia.** As for the genus, apart from: juxta oval to rectangular; valvae roughly rhombic; sacculus process claw-shaped, extending dorsad, parallel to or crossing with fibula; fibula slightly curved, spine-like, extending dorsad; posteriodorsal phallus apodeme with a small oval or semicircular sclerite, posterioventral apodeme with simple rodlike process.

**Female genitalia.** As for the genus, apart from: anterior antrum shortly coiled in coronal plane, with the exoculticle exhibiting a longitudinal sclerotized strip bearing transverse ridges (Fig. 33); sternite 8 intruding into the posteriorly somewhat constricted antrum, giving it a globular appearence.

#### Immature stages.

**Larva.** Only one specimen available, examined live. Black spots present on dorsal pinacula; in the final instar, MSD1 and MSD2 on a shared pinaculum on both meso- and metathorax; crochets mesally triordinal.

#### Etymology.

Composition of greek *pseud(o)* ‘false’ and *orbonalis*, meaning ‘false *orbonalis*’, referring to the similarities in external and male genital characters with *Leucinodes orbonalis*.

#### Distribution.

Angola, Senegal, Uganda.

#### Foodplants.

Solanaceae: *Solanum aethiopicum* L., *Solanum melongena* L.

#### Remarks.

We found this species among material from Senegal, Uganda and Angola, leaving a considerable distribution gap in Central Africa.

Recently, several interceptions of larvae in solanaceous fruits imported from Uganda have been recorded in England (own observation) and the Netherlands (Marja van der Straten, pers. comm.). *Leucinodes pseudorbonalis* is one of the three African *Leucinodes* species intercepted at European ports of entry.

### Leucinodes kenyensis sp. n.

Taxon classificationAnimaliaLepidopteraCrambidae

http://zoobank.org/C96D9272-2CEB-4916-948E-72C2999F35C3

Figs 5 , 18 , 26 , 34

#### Type-locality.

Kenya, Eastern Province, Marsabit District, Marsabit National Park Forest, 1158 m, 2°13.996'N, 37°55.676'E, 29 December 2003, R. S. Copeland leg.

#### Material examined.

**Type-specimens.** Holotype 1♂ “Kenya: Marsabit National | Park Forest. 1158 m. | 2°13.996'N 37°55.676'E. | 29 Dec 2003, A&M Coll. #2636 | R.S. Copeland; ICIPE/USDA”, “Reared from fruit: | Withania somnifera”, DNA Barcode “USNM ENT 007/19337”, “1133 | Nuss prep. no.”, coll. NMK. Paratypes 1♂, 1♀, same data, DNA Barcodes USNM ENT 719338, 719339, Nuss prep. no. 1135 (1♀ NMK, 1♂ SMTD); 1♀ Kenya, Laikipia Plateau, Mpala Research Centre, 0.293°N 36.899°E, 1650 m, 21.–24.vi.2005, leg. S.E. Miller, DNA Barcode USNM ENT 719976, Nuss prep. no. 1134 (USNM). – **Additional material. ZIMBABWE.** 1♂ Mashonaland, leg. H. B. Dobbie, prep. RM694 (BMNH).

#### Diagnosis.

Frons less strongly bulged than in *Leucinodes orbonalis*. Wing pattern indistinguishable from those of *Leucinodes orbonalis*, *Leucinodes africensis*, *Leucinodes rimavallis*, *Leucinodes pseudorbonalis* and *Leucinodes* spp., but distinguished from *Leucinodes malawiensis* by the absence of the forewing basal transversal streak and the presence of the apical half moon-shaped patch, and from *Leucinodes laisalis*, *Leucinodes ethiopica* and *Leucinodes ugandensis* by the predominantly white forewing ground colour. Distinguished in male genitalia from all other *Leucinodes* species except “*Leucinodes* spp.” (see below) by the prominent distal sacculus process arching anteriodorsally above the shorter, conical fibula. Distinguished from *Leucinodes* spp. by the more or less bulged subapical portion of costa (straight in *Leucinodes* spp.) and the weakly sclerotized basal section of the sacculus process (between sacculus and distal hook-shaped process) which is as wide as base of hook-shaped process (strongly sclerotized and narrower than base of hook-shaped process in *Leucinodes* spp.). Female genitalia resemble those of *Leucinodes rimavallis* in having an anteriad triangular process on each side of sternite 8 extending into the lateral antrum pockets, but the exocuticle of the anterior antrum forms a sclerotized tube (short sclerotized section in *Leucinodes rimavallis*).

#### Description of adults.

**Head.** As for the genus, with frons slightly bulged.

**Thorax.** As for the genus, with dorsal side brown.

**Wings.** Forewing length ♂ 9.0 mm, ♀ 9.0 mm; wing pattern as in *Leucinodes orbonalis*.

**Abdomen.** First segment whitish, remainder light to dark brown.

**Male genitalia.** As for the genus, apart from: saccus bent posteriodorsad (not an artifact of embedding); juxta oval to rectangular; valvae roughly triangular; costa subapically more or less bulged; ventral valva apex flipped over, covered with small tubercles; ventral valva edge smoothly rounded at sacculus; base of sacculus process broad, weakly sclerotized, leading over to a large, strongly sclerotized hook which encompasses the fibula dorsally; fibula conical, slightly curved, its base somewhat constricted, projecting dorsad; phallus similar to *Leucinodes orbonalis*, posteriodorsal apodeme with short dentate sclerite (sometimes indistinct), vesica with area of minute teeth.

**Female genitalia.** As for the genus, apart from: anterior antrum with tubular sclerotized exocuticle; sclerotized wall at antero-ventral edge of the ostium bursae which at rest closes the ostium bursae against abdominal segment 8. This wall is not melanized and can be stained with chlorazol black. It is delimited dorso-laterally by sclerotized and melanized lobes arising from the anterior edge of segment 8 just ventral of the apophyses anteriores. Anterior to the sclerotized wall there is a small, melanized colliculum.

#### Etymology.

The species is named after Kenya, the only country from where it is confidently recorded so far.

#### Distribution.

So far only known from Kenya. The record from Zimbabwe needs confirmation by investigation of female specimens and molecular analysis.

#### Foodplants.

Solanaceae: *Withania somnifera* (L.) Dunal.

#### Remarks.

There are further male specimens with indistinctive wing pattern and very similar genitalia, but DNA Barcode data suggest that among them are at least two further species. For more information, see under *Leucinodes* spp.

### Leucinodes malawiensis sp. n.

Taxon classificationAnimaliaLepidopteraCrambidae

http://zoobank.org/FECB8543-F50F-4CB3-A3D7-5C9ABAA3D9CC

Figs 6 , 19

#### Type-locality.

Malawi, Central Region, Lilongwe District, Ntchisi Forest Reserve, 1560 m, 13°18.99972'S, 34°02.99934'E, 18 February 2004, L. Aarvik leg.

#### Material examined.

**Type-specimen.** Holotype ♂ “MALAWI Central | Region, Lilongwe District: | Ntchisi Forest Reserve | 1560 m 18. ii. 2004 | leg. L. Aarvik”, DNA voucher SMTD Lep1617, prep. RM683 (NHMO).

#### Diagnosis.

Distinguished from the other *Leucinodes* species by the dark, straight-framed forewing base and the absence of the subapical mark of the forewing termen. The male genitalia are similar to those of *Leucinodes ethiopica* and *Leucinodes ugandensis*, but are distinct in the long spinoid process of the posterior phallus apodeme.

#### Description of adults.

**Head.** As for the genus, with frons flat.

**Thorax.** As for the genus, with dorsal side brown.

**Wings.** Forewing length ♂ 8.5 mm; forewing base dark brown, its outer margin a straight diagonal line from the costa to the maculation of the central hind margin, a triangular patch leading lateroposteriad from the costa with the costal half reddish-brown and the central tip white; outer median area with a faint brownish transverse streak; subterminal line indistinct except subapical thickening; apex white; hindwing antemedial line indistinct; dark discal spot; postmedial line clear at costal margin, fading out posteriad; anterior distal area with a faint brownish transverse streak; terminal wing veins dark-spotted.

**Abdomen.** Pale ochreous, first abdominal segment white, terminal segments brown.

**Male genitalia.** As for the genus, apart from: saccus elongated, U-shaped; juxta short, oval, twice as broad as long; valvae slender, tapering towards the dorsad bent apex; fibula small, triangular, oriented ventrad; sacculus process absent; phallus with a spinoid posteriad sclerotization emerging from the ventroposterior apodeme.

**Female genitalia.** Unknown.

#### Etymology.

Latinized *malawiensis* from the country Malawi where the holotype originates.

#### Distribution.

Malawi.

#### Foodplants.

Unknown.

#### Remarks.

*Leucinodes malawiensis* resembles species of the Neotropical genus *Neoleucinodes* Capps, 1948: It shares the prominent diagonal line in the forewing base with *Neoleucinodes dissolvens* (Dyar, 1914), but lacks the long, sabre-like cornutus in the phallus. The absence of the half moon-shaped pattern at the anterior half of the forewing’s outer margin is also found in *Proleucinodes* Capps, 1948. In the COI Barcode Neighbor Joining tree *Leucinodes malawiensis* clusters with *Neoleucinodes*, but is weakly supported with 50% Bootstrap support.

### Leucinodes laisalis

Taxon classificationAnimaliaLepidopteraCrambidae

(Walker, 1859) comb. n.

Figs 7–8 , 20 , 28 , 35 , 40–41 , 44–46

Megaphysa laisalis Walker 1859: 382–383. (Hampson 1899: 275 to *Sceliodes*)
Daraba idmonealis Walker 1859: 385–386.
Hyamia subterminalis Walker 1866: 1145.
Daraba plenisignata Walker 1866: 1977–1978.
Leucinodes translucidalis Gaede 1917: 398, **syn. n.**

#### Type-locality.

South Africa, Cape of Good Hope

#### Material examined.

**Type-specimen.** Holotype, sex unknown, [round white label with green border] “Type”; [round pale white label] [front] “C. G. | Hope”, [back] “44 | 6”; [rectangular blue label] “Megaphysa | laisalis Wlk | Type” (BMNH). – **Additional material.** **MOROCCO.** 1♂ Oued Cherrat, 17.ix.1952, leg. Ch. Rungs, prep. RM 734 (MNHN); **SENEGAL.** 1♂ Dakar, viii.1952, leg. A. Villiers, prep. RM736 (MNHN); **CÔTE D’IVOIRE.** 1♂ Bouaké, Inepa, 29.–30.xii.1983, leg. Stam, prep. RM688 (RMCA); **NIGERIA.** 1 ex. Oyo, Ibadan, International Institute of Tropical Agriculture, 7.501N 3.906E, 240m, 19.vi.2006, leg. G.M. Miller & T.M. Kuklenski, DNA Barcode USNM ENT 676643 (USNM); **KENYA.** 1♂ Nairobi, Kiambu (Kyambu), 6000 ft, x.1916, leg. H. L. Andrewes, BMNH Pyralidae slide No. 23136 (BMNH); 1♂ 1♀ Kitui (Katoteni), 30.viii.2005, ex larva on *Solanum incanum*, adult 30.ix.2005, leg. Muli & Okuku, preps RM301 & RM308 (SMTD); 10ex. Laikipia County, Laikipia Plateau, Mpala Research Centre, 0.293N 36.899E, 1650m, 13.ii.1999 (1ex.), 20.iv.1999 (4ex.), 17.vii.1999 (3ex.), 23.xii.1999 (1ex.), 06.xii.2002 (1ex.), leg. S.E. Miller & T.M. Kuklenski (5ex.), S.E. Miller & R. O’Meara (4ex.), S.E. Miller (1ex.), DNA Barcodes USNM ENT 196697–196706 (USNM); 1ex. Matthews Range, 1.24N 37.29E, 1506m, 17.i.2004, leg. R.S. Copeland, DNA Barcode USNM ENT 719748 (USNM); **TANZANIA.** 1♀ Morogoro, Sokoine University Garden, 06°50'S 037°38'E, 05.vii.2009, leg. J. & W. De Prins, prep. RM668 (RMCA); 1♂ Morogoro Distr. & Town, 550–600 m, 14.xi.1992, leg. L. Aarvik, prep. RM686 (coll. Aarvik); **SOUTH AFRICA.** 1♂ Mpumalanga, Barberton, northern edge of town, suburban garden- and bushland, 610 m, 15./16.i.2007, leg. T. Karisch, DNA Barcode BC MTD 01819, prep. RM504 (coll. Karisch); **SPAIN.** 1♂ Cadiz, 3 km NW Tarifa, 5 m, 25.ix.1987, leg. P. Skou, prep. RM380 (ZMUC); **GREAT BRITAIN (IMPORT).** see Suppl. material 2 (Fera material).

#### Diagnosis.

Distinguished from most other members of *Leucinodes* by the orange-brown to greyish forewing colour. Distinguished from *Leucinodes ethiopica* by the generally darker forewing colour with less amount of white. Differs from both *Leucinodes ethiopica* and *Leucinodes ugandensis* in: male genitalia with large, oval sacculus; broad, strongly sclerotized ventrad fibula; saccus well elongated; phallus coecum keeled, posteriodorsal apodeme with slim, fingerlike, well sclerotized process; female genitalia with antrum broad, its anterior end coiled, with exocuticle diffusely sclerotized.

#### Redescription of adults.

**Head.** Frons slightly bulged; labial palps upturned, greyish to brown, first meron on ventral side with forward-directed tuft, third meron in males half as long as second meron, considerably longer in females; maxillary palps minute; haustellum well developed; eyes large, hemispherical; ocelli present; antennae ciliate, cilia considerably longer in males; vertex with creamy white scales; chaetosemata absent.

**Thorax.** Dorsal side brown with greyish and dark brown scales mixed in; ventral side grey to whitish; legs predominantly whitish or grey, epiphysis present; tibial spurs 0, 2, 4 with outer spur 2/3 the length of inner spur.

**Wings.** Forewing length 7.0–11.5 mm, the females being somewhat larger; both sexes with one frenular bristle; forewing ground colour orange- to grey-brown, with the general *Sceliodes* wing pattern.

**Abdomen.** First segment whitish, remainder light-brown; older specimens often with darkened abdomen due to degeneration of abdominal fat body.

**Male genitalia.** As for the genus, apart from: vinculum saccus conspicuously elongated anteriad; juxta usually with small notch at median base; valvae emerging in narrow angle from vinculum; phallus with keeled coecum, posteriodorsal apodeme with slim, fingerlike, slightly curved and well sclerotized process, vesica with a short line of tiny cornuti.

**Female genitalia.** As for the genus, apart from: antrum long, tubular, anterior end coiled; apophysis pairs straight, apophyses anteriores with somewhat broader muscle attachment area at posterior quarter.

#### Distribution.

In Africa known from Côte d’Ivoire, Ghana, Kenya, Morocco, Nigeria, Senegal, South Africa, Tanzania (own observations). Externally, *Leucinodes laisalis* is similar to *Leucinodes ugandensis* (see below), therefore literature records from other African countries than those listed here need verification. In Europe recorded from Spain, Portugal (Speidel 1996, Huertas Dionisio 2000; own observations) and Great Britain (own observations). The records from Great Britain certainly refer to interceptions and it is assumed that those from the Iberian Penninsula also do not refer to a native occurrence. Huertas Dionisio (2000) recorded the species from *Solanum linnaeanum*, a species native to southern Africa.

#### Food plants.

Solanaceae: *Solanum incanum* L. (Kenya, leg. Muli & Okuku 2005), *Solanum anguivi* Lam. (“*Solanum sodomaeum* L.,”), *Solanum macrocarpon* L., *Solanum melongena* L., *Solanum linnaeanum* Hepper & P.-M. Jaeger, *Solanum lycopersicon* L. and *Capsicum annuum* L. (Hayden et al. 2013).

#### Immature stages.

**Larva.** Generally very similar to *Leucinodes orbonalis* and *Leucinodes africensis*. On the metathorax the MSD setae are usually on separate pinacula, while the mesothoracic MSD setae are usually on the same pinaculum. The abdominal D1 pinacula are often smaller than those of *Leucinodes orbonalis* and *Leucinodes africensis*, and lack dark spots or unpigmented areas (see Ogunwolu (1978) for a detailed larval description and chaetotaxy). **Pupa.** length ca. 8.5 mm; distal margins of cremaster usually evenly rounded, without distinct corners; spinulation of cremaster’s dorsal surface a little coarser than dorsal spinulation on abdominal segment 9 dorsal surface, no distinct small spines as in *Leucinodes orbonalis* or *Leucinodes africensis*; cocoon of beige coloured silk that does not darken significantly over time.

#### Remarks.

**Figure 47.**
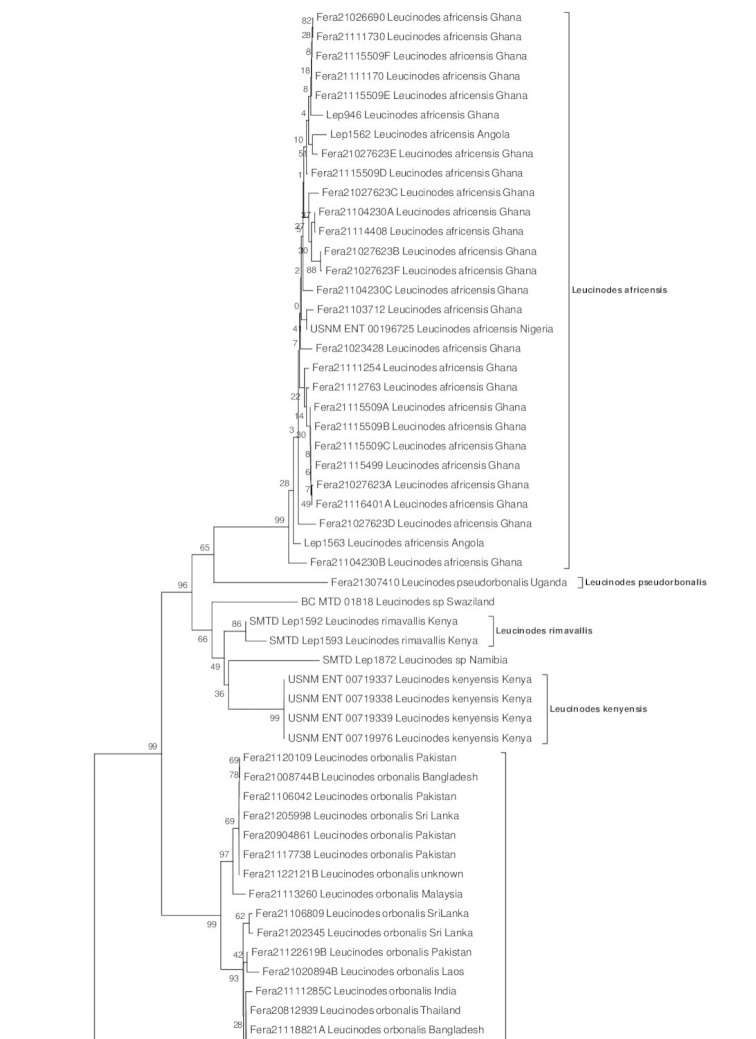

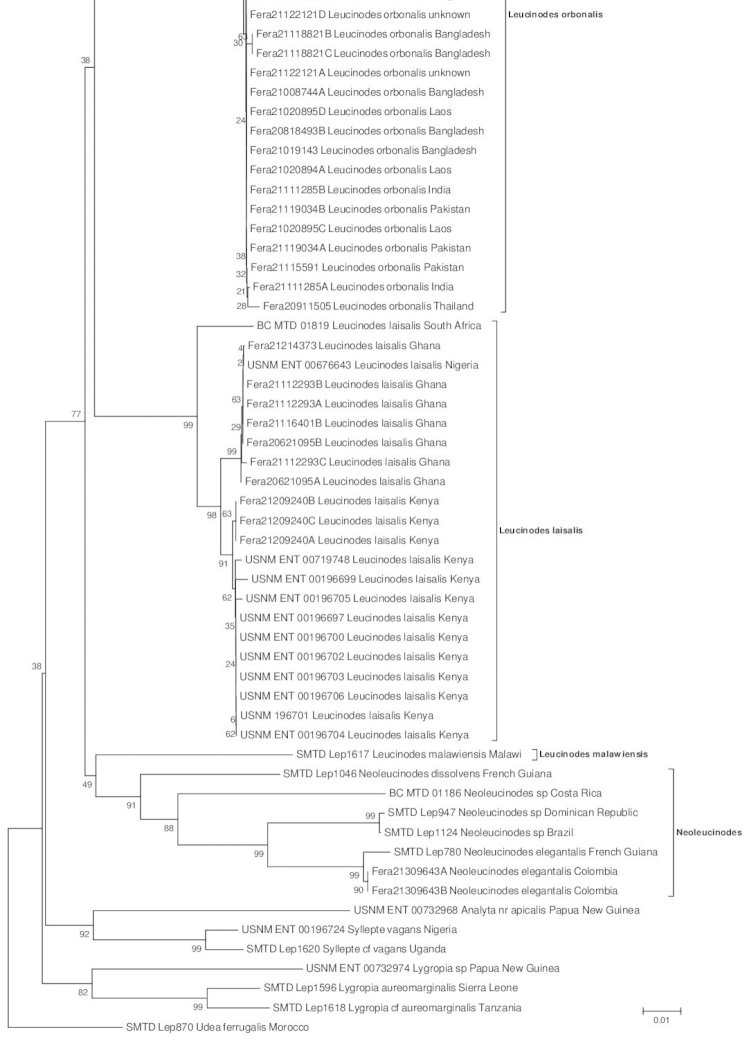
Neighbor Joining tree of COI Barcodes based on uncorr-p distances and rooted with *Udea ferrugalis*. Bootstrap support values derived from 1,000 Bootstrap replicates; scale bar represents 1% uncorr-p Barcode divergence.

We found a significant DNA Barcode difference of 2.4–2.8% uncorrected-p distance between the single South African specimen and the Kenyan and Ghanan/Nigerian Barcode clusters (Fig. 47; see also section ‘DNA Barcoding’ below). These differences in the DNA Barcode are not reflected in a divergent morphology of the clusters.

The record of *Leucinodes laisalis* from Belgium by Nyst (2004) is most probably a misidentification, since the illustrated imago resembles much more the whitish *Leucinodes* species. Apart from that, there is a European record of *Leucinodes laisalis* from Spain. Additionally, it is frequently intercepted with the import of solanaceous fruits in Great Britain.

Despite repeated search in the collection of the ZMHB, original material of *Leucinodes translucidalis* Gaede, 1917 from Tanzania, Tendaguru, could not be traced. According to the original description, this taxon can be regarded as conspecific with *Leucinodes laisalis* due to all details given in the original description. Especially the white triangle at the anterior line, another white triangle, though often somewhat inconspicuous, at the middle of costa, and the dark brown area below apex support the conspecifity with *Leucinodes laisalis*.

### Leucinodes ethiopica sp. n.

Taxon classificationAnimaliaLepidopteraCrambidae

http://zoobank.org/CC7E081A-7D4A-4C07-887F-4698DD8650EC

Figs 9 , 21 , 29

#### Type-locality.

Ethiopia, Dire Dawa Region, Dire Dawa District, Dire Dawa, December 1934, H. Ulenhuth leg.

#### Material examined.

**Type-specimens.** Holotype ♂ [red-circled label] “Holo- | type”, “Dire Daoua, | Abyssinia, | December 1934. | (H. Uhlenhuth).”; 19 paratypes: 11♂ 8♀ same data as holotype, including one with BM Pyralidae | slide 23138♂ (BMNH). – **Additional material.** **ERITREA.** Asmara, 20.x.1905. leg. N. Beccari (without abdomen), 1♀ same data except 28.i.1905 (BMNH); **ETHIOPIA.** 34 ex. same data as holotype except ii.1935, 4 ex. ditto except iv.1935, 7 ex. ditto except v.1935 including BM Pyralidae| slide 23139♀, 1 ditto except ix.1935, 2 ex. labelled Durleti [= Daleti] (BMNH); **SAUDI ARABIA.** 2♀ Taif (BMNH).

#### Diagnosis.

This species’ forewing colour has more ochreous than the whitish species of *Leucinodes* but less orange-brown to greyish than in *Leucinodes laisalis* and *Leucinodes ugandensis*. From *Leucinodes ugandensis* and *Leucinodes laisalis* it can be distinguished by the genitalia: in the male genitalia the transtilla arms each bear a dorsad spine; in the female genitalia the ductus bursae lacks the fine granular sclerotization, the antrum is strongly sclerotized, tubular and widest at its anterior end, and the oval ostial sclerites in the lateral antrum pockets are larger.

#### Description of adults.

**Head.** Head and appendages pale ochreous.

**Thorax.** Pale ochreous.

**Wings.** Forewing length 6.0–8.0 mm. Forewing mixed ochreous and white, an oblique dark ochreous fascia from above dorsum reaching halfway across wing, a blackish crescent before ochreous subterminal line, black dots along termen. Hindwing white, a small black discal spot, a faint irregular dark subterminal line, ochreous suffusion in outer part of wing in middle and towards apex.

**Abdomen.** Pale ochreous, first abdominal segment white.

**Male genitalia.** As for the genus, apart from: transtilla with short central notch, each transtilla arm with a dorsad spine; vinculum saccus with rounded tip; juxta oval to rectangular, apex with a short central notch; apex of valvae dorsally curved; small, spine-like dorsad fibula emerging from the central inner side of valva; sacculus with a fibula-like spiny dorsad process emerging ventrodistally of the fibula; posteriodorsal phallus apodeme with semicircular dentate sclerite.

**Female genitalia.** As for the genus, apart from: ductus bursae with fine longitudinal ripples; antrum tubular, with broader anterior end; apophyses anteriores at posterior half laterally broadened for muscle attachment.

#### Etymology.

Latinized *ethiopica* from the country Ethiopia where the holotype originates.

#### Distribution.

Eritrea, Ethiopia, Saudi Arabia.

#### Foodplants.

Unknown.

#### Remarks.

No COI Barcode sequences were obtained for this species.

### Leucinodes ugandensis sp. n.

Taxon classificationAnimaliaLepidopteraCrambidae

http://zoobank.org/533D9189-05DB-451D-ADB9-71AB3FD3DDD7

Figs 10 , 22 , 30

#### Type-locality.

Uganda, Eastern Uganda Region, Serere District, Okulongo, 8 December 1958, W. R. Ingram leg.

#### Material examined.

**Type-specimens.** Holotype ♂ [red-circled label] “Holo- | type”, “[transversally written] 1608 | “SERERE | Okulongo | 8 Dec. 1958 | W.R.Ingram | ex *Solanum* sp.”, “Pres. by | Com Inst Ent | BM1959 – 499”, “C.I.E. No. 16499”, with cocoon under specimen. 2 paratypes: 1♂ same data as holotype and “Pyralidae | Brit Mus | Slide No. | 14696”. 1♀ same data as holotype except 9 Dec. 1958 (BMNH). – **Additional material.** **ETHIOPIA.** 2♂ Diredaua, n.w. of Harar, 1914, leg. G. Kristensen, 1♀ Dire Dawa, Abyssinia, i.1935, leg. H. Uhlenkuth (BMNH); **SOUTH SUDAN.** 1♀ Tambura, Southern Bahr-al-Ghasal (BMNH); **SOMALIA.** 4♂ 1♀ Mogadishu, 17.–26.xi.1985, 7.vii.1986, 19–20.viii.1986 and 23.vii.1986, leg. A.G. Parker, BMNH Pyralidae slides No. 23140 & 23137 (BMNH); 1♂ Hargeison, 4300ft, v.1939, leg. M. Portal Hyatt (BMNH); **KENYA.** 1♀ Somaliland, Mandera, 47km SW of Hubera, 3000ft, 13.xi.1908, leg. W. Feather (BMNH).

#### Diagnosis.

Distinguished from the whitish species of *Leucinodes* and *Leucinodes ethiopica* by the predominantly brown forewing ground colour with minor white patches. Distinguished from *Leucinodes laisalis* in the male genitalia: less strongly sclerotized, valvae triangular, fibula small, tooth-like, a similarly shaped distal sacculus process present, saccus process shorter, phallus much shorter, dorsoposterior apodeme without slim, finger-like process.

#### Description of adults.

**Head.** Head and appendages pale fuscous, labial palpus short, erect.

**Thorax.** Pale fuscous, metathorax blackish fuscous.

**Wings.** Forewing length 6.5–11.5 mm. Forewing pale fuscous, an oblique brown partial fascia at halfway with white markings on either side, an orange triangle on dorsum beyond halfway, apex deep brown, separated by a whitish line. Hindwing whitish, fuscous suffused near margin in middle and towards apex, a faint subterminal line in costal part of wing.

**Abdomen.** Abdomen first segment whitish, remainder orange-brown.

**Male genitalia.** As for the genus, apart from: small, hooked dorsad fibula emerging from the ventrocentral inner side of valva; distal sacculus with dorsad ridge forming a bulge, followed by a spiny, curved terminal process overlapping with the fibula; phallus vesica with several small spiny cornuti.

**Female genitalia.** As for the genus, apart from: diffusely sclerotized exocuticula reaching into posterior ductus bursae; lateral antrum pockets rather small.

#### Etymology.

Latinized *ugandensis* from the country Uganda where the type specimens originate.

#### Distribution.

Ethiopia, Kenya, Somalia, South Sudan, Uganda.

#### Foodplants.

Solanaceae: *Solanum* sp.

#### Remarks.

No COI Barcode sequences were obtained for this species.

### Leucinodes spp.

Taxon classificationAnimaliaLepidopteraCrambidae

#### Note.

The following material contains male specimens from southern Africa with indistinctive wing pattern and very similar male genitalia to *Leucinodes kenyensis*. According to morphology, we could not separate these specimens from *Leucinodes kenyensis*. In contrast to the morphological data, two of these specimens, the single records from Namibia and Swaziland, have distinctive DNA Barcodes from *Leucinodes kenyensis* as well as from each other (Fig. 47). For the remaining specimens listed below, we did not obtain DNA Barcodes. In spite of the absence of further specimens for comparison, especially females, and the lack of convincing morphological differences, we are not going to describe these possibly distinct species here. This complex needs further study.

#### Material examined.

**KENYA.** 1♂ Rift Valley, Naivasha, 1900m, 0°46'56"S 36°25'23"E, 5.xii.2011, leg. D.J.L. Agassiz, prep. DJLA 1318 (coll. DJLA); **ZAMBIA.** 1♂ Chiwefwe, ii.1950, leg. N. Mitton, BMNH Pyralidae slide No. 23133 (BMNH); **NAMIBIA.** 1♂ Namibia, Waterberg, 20°30'S 17°14'E, 13.iii.2010, leg. F. Koch, DNA voucher MTD Lep1872, prep. RM708 (ZMHB); **SOUTH AFRICA.** 1♂ Cape Province, Knysna, Wilderness, iv.1950, leg. H.B.D. Kettlewell, BM Pyralidae slide 23128 (BMNH); 1♂ Johannesburg, 21.iv.1906, leg. A.T. Cooke, BMNH Pyralidae slide No. 23144 (BMNH); 1♂ E. Cape Prov., Katberg, 4,000ft, 1.–15.i.1933, BMNH Pyralidae slide No. 23150 (BMNH); 1♂ KwaZulu-Natal, Estcourt, leg. J.M. Hutchinson, prep. RM779 (BMNH); 1♂ Eastern Cape [Pondoland], Port St. John, ix.1923, leg. R.E. Turner, prep. RM780 (BMNH); **SWAZILAND.** 1♂ Lebombo-Mountains, Ndzevane Area near Nsoko, Acacia-rich bushland at the foot of the Lebombo Mountains, 23.–24.i.2007, leg. T. Karisch, DNA Barcode BC MTD 01818, prep. RM502 (coll. MTD).

### Taxa transferred to *Leucinodes*

#### Leucinodes cordalis

Taxon classificationAnimaliaLepidopteraCrambidae

(Doubleday, 1843) comb. n. (Margaritia)

Leucinodes cordalis (Doubleday, 1843). Type locality: New Zealand.
Daraba extensalis Walker, 1866 (synonymised by Hampson 1899). Type locality: New Zealand, Auckland.
Eretria obsistalis Snellen, 1880 (synonymised by Hampson 1899). Type locality: Indonesia, Sulawesi [Celebes], Boelekomba; Bonthain.
Sceliodes mucidalis Guenée, 1854 (synonymised by Hampson 1899). Type locality: Australia.

##### Material examined.

Syntype *cordalis* ♂ [circular white label with light blue border] “SYN- | TYPE”, [circular white label] [front] “New | Zealand”, [back] “42 | 55”, [rectangular white label] “Margarita [sic!] | cordalis Doubld. | SYNTYPE | det. D.J. Carter, 1966”, transparent capsule containing the head (BMNH).

##### Remarks.

*Leucinodes cordalis* is known to occur in New Zealand, Australia, and Indonesia: Sulawesi (Snellen 1880, Dugdale 1988, Shaffer et al. 1996).

#### Leucinodes raondry

Taxon classificationAnimaliaLepidopteraCrambidae

(Viette, 1981) comb. n. (Daraba)

##### Type locality.

Madagascar, Sambirano, Tsaratanana, Haut Sambirano, Besanetrikely Valley.

##### Remarks.

This species was described in *Daraba* Walker, 1859, a genus that has previously been synonymised with *Sceliodes* Guenée, 1854 by Hampson (1899). The type specimen of *Leucinodes raondry* (Viette, 1981), **comb. n.** from Madagascar could not be traced at MNHN. According to the original description and the illustration of the species given therein (Viette 1981), this species agrees with the diagnostic wing pattern elements of *Leucinodes*, and we therefore consider it as correctly placed in this genus. It differs from *Leucinodes laisalis* in the larger size, the more ochreous grey tone and the reduced half moon-shaped subapical patch of the forewings (Viette 1981). None of the species described here as new have the prominent dark subapical patch in the hindwings of *Leucinodes raondry*, so that conspecifity with any of them can be ruled out.

#### Leucinodes grisealis

Taxon classificationAnimaliaLepidopteraCrambidae

(Kenrick, 1912) comb. n. (misplaced) (Sceliodes)

##### Type locality.

Indonesia, Dutch New Guinea [West Papua], Arfak Mountains, 4000 ft

##### Material examined.

Holotype ♂ [circular label with red border] “Type”, [rectangular whitish label, handwritten, first two words underlined] “Sceliodes | grisealis | Kenrick. | TYPE.”, [rectangular white label, handwritten] “SCELIODES | grisealis”, [rectangular greyish label] “Arfak Mountains, | North New Guinea. | 4,000ft. Feb.-Mar., 1909. | C.B.Pratt.”, [rectangular white label] “Kenrick Coll. | Brit.Mus. | 1928–34.” (BMNH). Female unknown.

##### Remarks.

Due to the synonymisation of *Sceliodes*, this species is provisionally transferred to *Leucinodes*, as no proper generic placement has been found. Compared to *Leucinodes*, several differences can be found in wing pattern of *grisealis* Kenrick, 1912: In the fore wing, the postmedian line is originating in the apex, and its median protrusion is closely approaching the termen; the half moon-shaped patch below apex is protruding beyond M_3_; in the hind wing, the postmedian line is originating closer to the apex and is running closer to the termen.

### Taxa excluded from *Leucinodes*

#### Analyta

Taxon classificationAnimaliaLepidopteraCrambidae

Lederer, 1863

Analyta Lederer, 1863. Type species: *Analyta albicillalis* Lederer, 1863.
Hyperanalyta Strand, 1918, **syn. rev.** Type species: *Analyta pseudoapicalis* Strand, 1918.
Analyta apicalis (Hampson, 1896), **comb. n.** (*Leucinodes*). Type locality: India, Dharamsala. Sri Lanka.
Analyta (Hyperanalyta) pseudoapicalis Strand, 1918 (synonymised by Shibuya 1928). Type locality: China, Taiwan, Anping.

##### Material examined.

Types: Holotype *albicillalis* [circular white label with red border] “Type”, [beige label with brown border and triangular edges] “Amboina | Doll.”, [rectangular white label] “Felder | Collection.”, [rectangular white label] “Rothschild | Bequest | B.M.1939-1.”, [rectangular beige handwritten label] “Amboina | Dol.”, [rectangular beige handwritten label] “Analyta | albicillalis m”, [rectangular brown label with central white area, roundly bordered by dark brown and yellow] “albicillalis Led.” (BMNH); Holotype *apicalis* ♂ [circular label with red border] “Type”, [rectangular white label] “4-94”, [rectangular white label] “Ceylon | 95-119”, [rectangular white handwritten label] “Leucinodes | apicalis | type ♂ Hmpsn.”, transparent capsule with abdomen (BMNH); Holotype *pseudoapicalis* ♂ [red rectangular label] “Holotypus”, [white rectangular label] “Anping | Formosa | H. Sauter VI.1911.”, [rectangular white label] “Analyta | pseudoapi | calis m.| Strand det. ♂” (SDEI).

##### Remarks.

*Hyperanalyta* Strand, 1918, with type species *Analyta pseudoapicalis* Strand, 1918 was established as a subgenus of *Analyta* Lederer, 1863. Later, *Analyta pseudoapicalis* was synonymised with *Leucinodes apicalis* Hampson, 1896 by Shibuya 1928. Thus, *Hyperanalyta* had to be regarded as a synonym of *Leucinodes*. Our investigation of *Leucinodes apicalis*, *Analyta pseudoapicalis* and *Analyta albicillalis* Lederer, 1863, the type species of *Analyta* Lederer, 1863, showed for all three species group taxa the presence of two frenular bristles in females (one in *Leucinodes*), the lack of distal discoidal stigma and L-shaped or triangular spot at central dorsum as characteristic for *Leucinodes*, but a homologous wing pattern common for the three taxa: forewing antemedian area brown; distal discoidal stigma a pale brown thin line; postmedian line sinuate; apical dark spot reaching outer forewing margin (white border in *Leucinodes*). We therefore rule out the congenerity of *Leucinodes apicalis* and *Analyta pseudoapicalis* with *Leucinodes* and transfer both to *Analyta*.

#### Lygropia

Taxon classificationAnimaliaLepidopteraCrambidae

Lederer, 1863

Lygropia Type species: *Asopia unicoloralis* Guenée, 1854.

#### Lygropia aureomarginalis

Taxon classificationAnimaliaLepidopteraCrambidae

(Gaede, 1916) comb. n. (Leucinodes)

##### Type locality.

Cameroon, Buea.

##### Material examined.

Holotype ♂ [small blue label] “Gr. Kamerunberg | Buea 1.–10. XI. 10 | 1000–1200 m | E. Hintz S. G.”, [large blue handwritten label with black border] “Leucinodes | aureomarginalis | 83:8a Type Gaede” (ZMHB).

##### Remarks.

*Lygropia* is a polyphyletic genus containing 62 species (Nuss et al. 2003–2014). We provisionally transfer *Lygropia aureomarginalis* (Gaede, 1916), **comb. n.** (*Leucinodes*) from Cameroon to this genus, as this species, according to wing pattern elements, is congeneric, if not conspecific, to *Lygropia vinanyalis* Viette, 1958 from Madagascar. *Lygropia aureomarginalis* can be distinguished externally from species of *Leucinodes* by the shiny golden wing maculation and the presence of two frenulum bristles in the female.

#### Syllepte

Taxon classificationAnimaliaLepidopteraCrambidae

Hübner, 1823

Syllepte Hübner, 1823. Type species: *Syllepte incomptalis* Hübner, 1823.
Syllepte hemichionalis (Mabille, 1900), **comb. rev.** (*Sylepta* [sic]). Type locality: Madagascar, baie d’Antongil
Syllepte hemichionalis idalis Viette, 1958, **comb. rev.** (*Syllepta* [sic]). Type locality: Comoros, Mohéli.
Syllepte vagans (Tutt, 1890), **comb. n.** (*Aphytoceros*). Type locality: Great Britain, Chepstow.
Aphytoceros longipalpis Warren, 1892 (synonymised by South 1897). Type locality. South Africa, Transvaal.

##### Material examined.

Types: Holotype *hemichionalis* ♂ [red label] “TYPE”, [pale green label] “MUSEUM PARIS | MADAGASCAR | BAIE D’ANTONGIL | A. MOCQUERYS 1898”, [whitish handwritten label] “Sylepta | hemichionalis Mab. | Ann.Soc.Ent.Fr. | 1899 p. 745.”, [whitish handwritten label] “Sylepta | hemichionalis | Mab.” (MNHN); Holotype subsp. *idalis* ♂ [red label] “TYPE”, [white label] “COMORES | MOHÉLI XI–1955 | A. ROBINSON”, [white handwritten label] “[underlined] Sylepta ♂ | [underlined] hemichionalis | [underlined] idalis n. subsp. | Holotype [underlined] P.Viette” (MNHN); Holotype syn. *longipalpis* ♀ [circular label with red border] “Type”, [white label] “Transvaal | 91–21” (BMNH). The whereabouts of the type specimen of *vagans* Tutt, 1890 are not known.

##### Remarks.

*Syllepte hemichionalis* and *Syllepte vagans* are similar to each other in wing pattern elements, the presence of two frenular bristles in the female hindwings and features of male genitalia (see Suppl. material 2I), but not so to *Leucinodes*. Their wing pattern differs from *Leucinodes* in the following: forewing without the L-shaped or triangular spot at central dorsum of *Leucinodes* and without the half moon-shaped subapical patch; postmedian line broad, straight, but with long loop before dorsum, reaching to central part of forewing; dorsum with broad brown line leading from brown antemedian area to distal end of postmedian line; in the hindwing they have a comma- or V-shaped distal discoidal sigma, light brown with dark brown border. This homologous wing pattern is also found in *Syllepte chalybifascia* Hampson, 1896 from India and *Syllepte dottoalis* Schaus, 1927 from the Philippines, and we consider them congeneric with each other as well as with *Syllepte hemichionalis* and *Syllepte vagans*. The genus *Syllepte* comprises 193 species (Nuss et al. 2003–2014), is probably polyphyletic and in need for taxonomic revision.

#### Deanolis

Taxon classificationAnimaliaLepidopteraCrambidae

Snellen, 1899

Deanolis Type species: *Deanolis sublimbalis* Snellen, 1899

#### Deanolis iriocapna

Taxon classificationAnimaliaLepidopteraCrambidae

(Meyrick, 1938) comb. n. (Sceliodes)

##### Type locality.

Indonesia, Yogyakarta

##### Material examined.

Holotype ♂ [circular label with red circle] “Holo- | type”, [white label] “südl. M.-Java | Djokjakarta | H.Overbeck”, [white label] “M600”, [white label] “Sceliodes | iriocapna Meyr. | det. E. Meyrick.”, [white label] “Sceliodes | iriocapna | 1/1 | Meyrick | E.Meyrick det. | in Meyrick Coll.”, [white label] “Meyrick Coll. | B.M. 1938-290.” (BMNH).

##### Remarks.

The wing pattern of *iriocapna* Meyrick, 1938 exhibits none of the features found in *Leucinodes*. Instead, the fore wings are pale yellow, with a yellowish costa, a dark spot in both outer edges of the cell, and a reddish undulating margin along the termen. The hind wings are of the same pale yellow ground colour, and the anterior half of the termen exhibits a similar margin as found in the fore wings. This wing pattern is common to the genus *Deanolis* Snellen, 1899, where this species is correctly placed (pers. comm. James E. Hayden).

### Identification key for African *Leucinodes* species based on male genitalia:

**Table:** 

1	Transtilla arms each with a dorsad spine	***Leucinodes ethiopica* sp. n.**
1*	Genitalia without these structures	**2**
2	Sacculus without distal process; posterior phallus apodeme with finger-like process	**3**
2*	Distal process of sacculus present; posterior phallus apodeme without finger-like process	**4**
3	Fibula broad, strongly sclerotized, emerging just ventral of costa; sacculus more than half the length of valva; saccus very elongated, tip pointed; dorsal posterior phallus apodeme with slim, finger-like, well sclerotized process	***Leucinodes laisalis* (Walker, 1859), comb. n.**
3*	Fibula small, strongly sclerotized, emerging near ventral valva edge; sacculus less than half the length of valva; saccus elongated, tip not pointed; ventral posterior phallus apodeme with slim, posterioventrally protruding, well sclerotized process	***Leucinodes malawiensis* sp. n.**
4	Distal sacculus process projecting towards valva apex, straight or apically hooked, sometimes branching, apex dentate; juxta subulate; posterior phallus apodeme with oval saw blade-shaped sclerotization	**5**
4*	Distal sacculus process tooth- or hook-like; juxta much broader; posterior phallus apodeme without oval saw blade-shaped sclerotization	**6**
5	Valva apex rounded; ventrad fibula stout, with broad base, straight or slightly curved; distal sacculus process hook-shaped, not branching, spanning ≤ half the distance fibula base–valva apex	***Leucinodes rimavallis* sp. n.**
5*	Valva apex pointed; ventrad fibula slender, with narrower base, curved; distal sacculus process straight or hook-shaped, sometimes branching, spanning > half the distance fibula base–valva apex	***Leucinodes africensis* sp. n.**
6	Dorsad fibula conical and smaller than distal sacculus process, which is large, hook-like and arching above fibula	***Leucinodes kenyensis* sp. n.**
6*	Dorsad fibula and fibula-like sacculus process being roughly of same shape and size and running parallel or crossing each other	**7**
7	Fibula and fibula-like sacculus process large, spanning almost the entire valva transversally; ventral valva edge not straight but with arch dorsal of fibula and fibula-like process; posterior phallus apodeme ventrally with oval sclerite	***Leucinodes pseudorbonalis* sp. n.**
7*	Fibula and fibula-like sacculus process small, barely reaching the valva centre; ventral valva edge more or less straight; posterior phallus apodeme without oval sclerite	***Leucinodes ugandensis* sp. n.**

### DNA Barcoding

The Neighbor Joining (NJ) analysis based on uncorrected p-distances (Fig. 47) groups taxa which are excluded from *Leucinodes* in this study outside of the *Leucinodes* + *Neoleucinodes* group. *Deanolis iriocapna*, *Leucinodes ethiopica* and *Leucinodes ugandensis* have not been barcoded and are therefore not included in the NJ tree. The *Leucinodes* + *Neoleucinodes* group forms a polytomy comprising *Neoleucinodes*, *Leucinodes malawiensis*, *Leucinodes laisalis* and a group containing the remaining *Leucinodes* species. Within this last group, 11 barcode clusters are revealed.

*Leucinodes laisalis* clusters in two Barcode groups: One group containing all specimens imported with fruits from Kenya, Ghana and Nigeria, and a second group comprising a single South African specimen. This single specimen shows high uncorr-p distances of 2.4–2.8% to the other *Leucinodes laisalis* specimens.

*Leucinodes orbonalis* clusters in two groups, separated by 1.1–1.8% uncorr-p distance. Within-subcluster distances are 0–0.5% for the smaller and 0–0.9% for the larger of the two subclusters.

A polytomous cluster comprising *Leucinodes rimavallis*, *Leucinodes kenyensis* and two undescribed ‘Barcode species’ (*Leucinodes* spp.) are sister to (*Leucinodes africensis* + *Leucinodes pseudorbonalis*).

Barcode sharing among different *Leucinodes* morphospecies is not observed.

## Discussion

**Figure 48.**
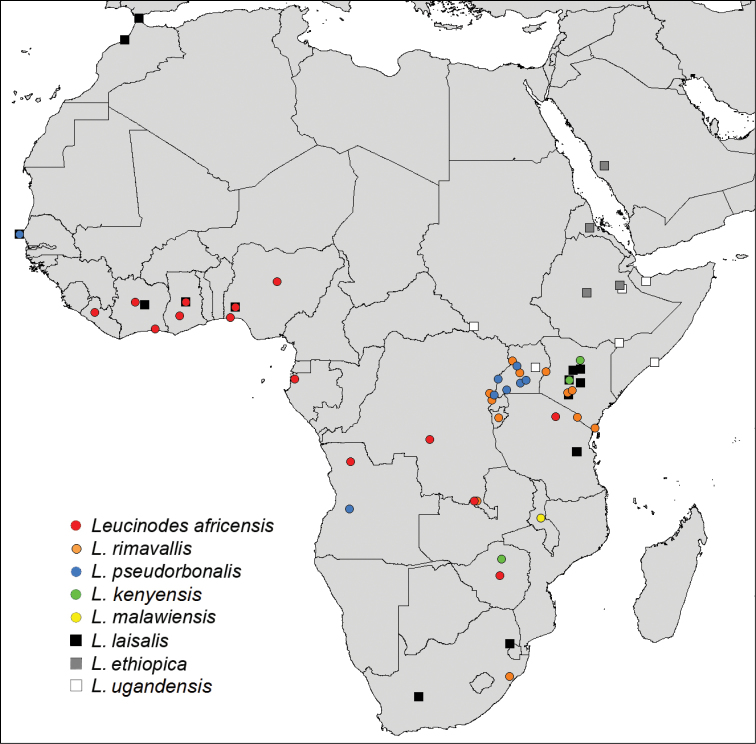
Distribution map of African *Leucinodes* species.

The investigation of the *Leucinodes* species from sub-Saharan Africa reveals a new picture on the taxonomy of the Solanaceae fruit feeders. Instead of one widely distributed species in Asia and Africa, there is an endemic species complex in Africa and *Leucinodes orbonalis* is restricted to Asia, corroborating the statement by Hayden et al. (2013). Among the African species, *Leucinodes africensis* and *Leucinodes laisalis* are widely distributed on the continent, whereas *Leucinodes ethiopica*, *Leucinodes ugandensis*, *Leucinodes rimavallis* and *Leucinodes kenyensis* seem to have a rather restricted range. *Leucinodes malawiensis* is only known from one specimen from Malawi. These data may indicate different, though partly overlapping patterns of distribution as seen in Fig. 48, but this is still an incomplete dataset, resulting in fragmentary distribution patterns for *Leucinodes pseudorbonalis* and *Leucinodes rimavallis*. The possibility of transport by man also has to be taken into consideration.

Food plant data are only available for *Leucinodes africensis*, *Leucinodes laisalis*, *Leucinodes pseudorbonalis*, *Leucinodes rimavallis* and *Leucinodes kenyensis* (Table 2). While *Leucinodes laisalis* is known to feed on a variety of cultivated and non-cultivated Solanaceae, the other four species are known from cultivated Solanaceae only. This may rather refer to a bias of investigation, which so far has concentrated on fruits of economic importance. In nature, the *Leucinodes* species may have different host plants to which they were originally adapted. Those wild hosts may also have certain areas of origin. Such a scenario has to be expected, considering the fact that several species of *Leucinodes* have evolved in Africa. Some of these species might be restricted to their wild host plants and unable to switch to other, cultivated Solanaceae species. In contrast, species able to adapt to cultivated Solanaceae might be transported with fruits in trade, resulting in a larger range within Africa.

**Table 1.** Uncorrected p-distances for the DNA-barcoded species of *Leucinodes*. Values in bold denote intraspecific distances, plain values represent interspecific distances.

	*orbonalis*	*africensis*	*rimavallis*	*pseudorbonalis*	*kenyensis*	*malawiensis*	*laisalis*

***orbonalis* (n=31)**	**0–1.8%**						
***africensis* (n=29)**	5.5–7.2%	**0–1.5%**					
***rimavallis* (n=2)**	4.2–5.7%	3.8–5.7%	**0.4%**				
***pseudorbonalis* (n=1)**	6.4–7.0%	5.4–6.2%	3.3–5.2%	**n.a.**			
***kenyensis* (n=4)**	5.3–6.0%	5.4–6.2%	2.2–2.4%	5.2%	**0%**		
***malawiensis* (n=1)**	9.6–10.7%	11.4–12.1%	10.0–10.8%	11.7%	11.2%	**n.a.**	
***laisalis* (n=22)**	7.3–8.7%	9.4–10.8%	7.8–8.9%	9.9–10.4%	9.4–9.8%	9.3–9.9%	**0–2.8%**

**Table 2.** Food plant records of African *Leucinodes* species. Cultivated plants are given in bold.

	*Leucinodes africensis*	*Leucinodes kenyensis*	*Leucinodes laisalis*	*Leucinodes pseudorbonalis*	*Leucinodes rimavallis*
***Capsicum annuum***			x		
***Solanum anguivi***			x		
***Solanum aethiopicum***	x			x	
*Solanum incanum*			x		
*Solanum linnaeanum*			x		
***Solanum lycopersicon***	x		x		
***Solanum macrocarpon***			x		
***Solanum melongena***	x		x	x	x
***Withania somnifera***		x			x

After movement of larvae with fruits in African trade, they may be more frequently intercepted by trade with those fruits into other continents. Most imports of *Leucinodes* specimens from Africa into Europe refer to *Leucinodes africensis*, which has been frequently imported with fruits during the last 50 years. In contrast, *Leucinodes laisalis* has been much less frequently recorded, and *Leucinodes pseudorbonalis* as well as *Leucinodes rimavallis* only very recently in fruit imports from Uganda. Since our investigations show that *Leucinodes orbonalis* does not occur in Africa, interceptions of *Leucinodes* from Africa into other continents will need to be re-investigated for their species identity and will likely require, at least in parts, revisions of the quarantine regulations. Furthermore, if *Leucinodes* species are transported in trade, it has to be considered that species of *Leucinodes* and related South American genera, e.g. *Euleucinodes* Capps, 1948, *Neoleucinodes* and *Proleucinodes*, might also become introduced from one to another of the southern continents.

Analysis of the COI gene of the *Leucinodes* species demonstrated that interspecific differences allow the use of the marker as a DNA barcode for species identification. However, for *Leucinodes kenyensis* and “*Leucinodes* spp.”, we found little morphological differences but two distinct barcode species within *Leucinodes* spp. Moreover, we observed high intraspecific divergence in *Leucinodes laisalis* with one specimen from South Africa exhibiting a COI distance of 2.8%.

Outside Africa, the taxonomy and phylogeny of *Leucinodes* requires further research. The characteristic forewing pattern elements of the Old World *Leucinodes* is also found in the New World genera *Euleucinodes*, *Neoleucinodes* and *Proleucinodes*, all described by Capps (1948). In male genitalia, *Leucinodes* is distinguished from these three genera by the absence of cornuti in the phallus, from *Euleucinodes* and *Proleucinodes* also by the presence of a fibula, and further from *Euleucinodes* by the dorsal location of the uncus spines. In female genitalia, *Leucinodes* is distinguished from *Proleucinodes* by the presence of lateral antrum pockets (condition unknown in *Euleucinodes*), but cannot be clearly distinguished from *Neoleucinodes*, in which antrum pockets can be absent (e.g., *Neoleucinodes elegantalis* (Guenée, 1854)) or present (*Neoleucinodes prophetica* (Dyar, 1914), *Neoleucinodes torvis* Capps, 1948), and pocket sclerites can be absent (e.g., *Neoleucinodes elegantalis*) or present (*Neoleucinodes imperialis* (Guenée, 1854), *Neoleucinodes torvis*). Whether these differences refer to a typological classification or justify the maintenance of the current generic classification needs to be investigated by phylogenetic analysis.

For Austral-Asia, there remain twelve nominal *Leucinodes* species (Nuss et al. 2003–2014). Other than *Leucinodes orbonalis*, at least some of these species are certainly misplaced in *Leucinodes*, e.g. *Leucinodes labefactalis* Swinhoe, 1904 from Borneo and *Leucinodes perlucidalis* Caradja, 1933 from China. Therefore, the Asian *Leucinodes* are in need of taxonomic revision. This also points to the question whether all *Leucinodes* samples intercepted from Asian exports refer to *Leucinodes orbonalis*, or whether there are several *Leucinodes* species of economic importance in Asia as well (Hayden et al. 2013, Chang et al. 2014, Gilligan and Passoa 2014).

Our work contributes to the identification of African *Leucinodes* species, based on adult characters and on DNA Barcodes, by which also the immature stages can be efficiently distinguished. This may help to systematically survey the continent for distribution of species, in order to discover their wild host plants and their movements in trade. At this stage of knowledge it wouldn’t be a surprise to discover additional, still unknown species. The results of our completely revised taxonomy of African *Leucinodes* suggests that a revision of the EPPO A1 List of pests recommended for regulation as quarantine pests (EPPO 2013) will be necessary. During the period from 2004 to 2007, 121 interceptions of *Solanum* fruits infested by *Leucinodes* were recorded by several EPPO member countries. The majority (94 consignments) originated from Thailand, Ghana accounted for 18 infested consignments (EPPO 2008). *Leucinodes orbonalis* is also ranked as quarantine species important for the USA, but specimens intercepted from Africa cannot be *Leucinodes orbonalis*, as stated by Hayden et al. (2013) and Gilligan and Passoa (2014) and as shown by our results. The USA has recorded 1745 interceptions from Ghana to the USA between 1985 and 2004 (Boateng et al. 2005).

## Conclusion

A careful revision of *Leucinodes* in sub-Saharan Africa resulted in the new synonymy of the genus *Sceliodes* syn. n., the revised synonymy of *Hyperanalyta* with *Analyta*, the transfer of four species to *Leucinodes*, the description of seven new *Leucinodes* species, the new synonymy of one species, the omission of the type species *Leucinodes orbonalis* from the African list and the generic transfer of five species found to be misplaced in *Leucinodes*. Of the eight species recognized from Africa now, at least four are frequently intercepted among imports of solanaceous fruits at European ports of entry. We provide the DNA Barcode for these four and two additional African *Leucinodes* species, allowing the identification of all life stages of these species.

Our work shows that typological concepts of taxonomy based on superficial similarity were still the state of the art in the genus *Leucinodes*. The discovery of a complex of highly similar species demonstrates that traditional morphological methods and DNA Barcoding are helpful tools to detect species diversity and to improve their classification based on sound arguments.

Similar revisionary work remains to be done for Austral-Asian *Leucinodes*. A phylogenetic study including *Leucinodes* and the New World *Euleucinodes*, *Neoleucinodes* and *Proleucinodes* is needed in order to test the monophyly of these genera (Hayden and Mally, in prep.).

## Supplementary Material

XML Treatment for Leucinodes

XML Treatment for Leucinodes orbonalis

XML Treatment for Leucinodes africensis

XML Treatment for Leucinodes rimavallis

XML Treatment for Leucinodes pseudorbonalis

XML Treatment for Leucinodes kenyensis

XML Treatment for Leucinodes malawiensis

XML Treatment for Leucinodes laisalis

XML Treatment for Leucinodes ethiopica

XML Treatment for Leucinodes ugandensis

XML Treatment for Leucinodes spp.

XML Treatment for Leucinodes cordalis

XML Treatment for Leucinodes raondry

XML Treatment for Leucinodes grisealis

XML Treatment for Analyta

XML Treatment for Lygropia

XML Treatment for Lygropia aureomarginalis

XML Treatment for Syllepte

XML Treatment for Deanolis

XML Treatment for Deanolis iriocapna

## Supplementary materials

Supplementary material 1 Overview of specimens intercepted in Great Britain by the Food and Environment Research Agency (Fera). Data type: sample list. This dataset is made available under the Open Database License (http://opendatacommons.org/licenses/odbl/1.0/). The Open Database License (ODbL) is a license agreement intended to allow users to freely share, modify, and use this Dataset while maintaining this same freedom for others, provided that the original source and author(s) are credited.Richard Mally, Anastasia Korycinska, David J. L. Agassiz, Jayne Hall, Jennifer Hodgetts, Matthias Nuss

Supplementary material 2 Overview of barcoded specimens. Data type: sample list. This dataset is made available under the Open Database License (http://opendatacommons.org/licenses/odbl/1.0/). The Open Database License (ODbL) is a license agreement intended to allow users to freely share, modify, and use this Dataset while maintaining this same freedom for others, provided that the original source and author(s) are credited.Richard Mally, Anastasia Korycinska, David J. L. Agassiz, Jayne Hall, Jennifer Hodgetts, Matthias Nuss
