# Supplementary material for: Discovery of an unknown diversity of Leucinodes species damaging Solanaceae fruits in sub-Saharan Africa and moving in trade (Insecta, Lepidoptera, Pyraloidea)
Source: Zookeys. 2015 Jan 19;(472):117–62. doi: 10.3897/zookeys.472.8781 (PMC4304033; doi:10.3897/zookeys.472.8781)
Supplement: Supplementary material 1 — Overview of specimens intercepted in Great Britain by the Food and Environment Research Agency (Fera). [file zookeys-472-117-s001.docx]

# Appendix II

**Overview of specimens intercepted in Great Britain by the Food and Environment Research Agency**

abbreviations: AP - airport; bp - base pairs; F - female; *L.* - *Leucinodes*; lv - larva; M - male; *N.* - *Neoleucinodes*; pu - pupa; *S.* - *Solanum*.

| **Species** | **Sex** | **Origin** | **Host** | **Plant Health and Seeds Inspector** | **Port of entry** | **Date intercepted** | **Unique Fera number** | **Barcode length [bp]** |
| --- | --- | --- | --- | --- | --- | --- | --- | --- |
| *L. africensis* | M | Ghana | *S. melongena* | Joanna Wyzkiewicz | London Heathrow AP | 18-XI-2010 | 21026690A | 601 |
| *L. africensis* | 1 lv | Ghana | *S. melongena* | Joanna Wyzkiewicz | London Heathrow AP | 18-XI-2010 | 21026690B | - |
| *L. africensis* | M | Ghana | *S. melongena* | Joanna Wyzkiewicz | London Heathrow AP | 30-XI-2010 | 21027623A | 598 |
| *L. africensis* | M | Ghana | *S. melongena* | Joanna Wyzkiewicz | London Heathrow AP | 30-XI-2010 | 21027623B | 618 |
| *L. africensis* | F | Ghana | *S. melongena* | Joanna Wyzkiewicz | London Heathrow AP | 30-XI-2010 | 21027623C | 616 |
| *L. africensis* | F | Ghana | *S. melongena* | Joanna Wyzkiewicz | London Heathrow AP | 30-XI-2010 | 21027623D | 610 |
| *L. africensis* | F | Ghana | *S. melongena* | Joanna Wyzkiewicz | London Heathrow AP | 30-XI-2010 | 21027623E | 605 |
| *L. africensis* | F | Ghana | *S. melongena* | Joanna Wyzkiewicz | London Heathrow AP | 30-XI-2010 | 21027623F | 601 |
| *L. africensis* | M | Ghana | *S. melongena* | Karen Freeman | London Heathrow AP | 21-VIII-2011 | 21115509A | 618 |
| *L. africensis* | M | Ghana | *S. melongena* | Karen Freeman | London Heathrow AP | 21-VIII-2011 | 21115509B | 617 |
| *L. africensis* | M | Ghana | *S. melongena* | Karen Freeman | London Heathrow AP | 21-VIII-2011 | 21115509C | 617 |
| *L. africensis* | F | Ghana | *S. melongena* | Karen Freeman | London Heathrow AP | 21-VIII-2011 | 21115509D | 610 |
| *L. africensis* | F | Ghana | *S. melongena* | Karen Freeman | London Heathrow AP | 21-VIII-2011 | 21115509E | 611 |
| *L. africensis* | F | Ghana | *S. melongena* | Karen Freeman | London Heathrow AP | 21-VIII-2011 | 21115509F | 610 |
| *L. africensis* | 2 lv | Ghana | *S. melongena* | Karen Freeman | London Heathrow AP | 21-VIII-2011 | 21115509G | - |
| *L. africensis* | 1 pu | Ghana | *S. melongena* | Karen Freeman | London Heathrow AP | 21-VIII-2011 | 21115509H | - |
| *L. africensis* | M | Ghana | *S. melongena* | Joanna Wyzkiewicz | London Heathrow AP | 03-III-2011 | 21104230A | 617 |
| *L. africensis* | M | Ghana | *S. melongena* | Joanna Wyzkiewicz | London Heathrow AP | 03-III-2011 | 21104230B | 616 |
| *L. africensis* | F | Ghana | *S. melongena* | Joanna Wyzkiewicz | London Heathrow AP | 03-III-2011 | 21104230C | - |
| *L. africensis* | F | Ghana | *S. melongena* | Adam Dollimore | London Heathrow AP | 15-VI-2011 | 21111170A | 610 |
| *L. africensis* | 1 lv | Ghana | *S. melongena* | Adam Dollimore | London Heathrow AP | 15-VI-2011 | 21111170B | - |
| *L. africensis* | M | Ghana | *S. melongena* | Nicholas Finnis | London Heathrow AP | 03-VIII-2011 | 21114408 | 610 |
| *L. africensis* | 2 lv | Ghana | *S. melongena* | Nicholas Finnis | London Heathrow AP | 03-VIII-2011 | 21114408 | - |
| *L. africensis* | M | Ghana | *S. melongena* | Simon Honey | London Heathrow AP | 20-VIII-2011 | 21115499 | 617 |
| *L. africensis* | M | Ghana | *S. melongena* | Nicholas Finnis | London Heathrow AP | 08-VI-2011 | 21110635 | - |
| *L. africensis* | M | Ghana | *S. melongena* | James Robson | London Heathrow AP | 23-VI-2011 | 21111730 | 601 |
| *L. africensis* | M | Ghana | *S. melongena* | Jason Pollock | London Heathrow AP | 24-II-2011 | 21103712 | 609 |
| *L. africensis* | F | Ghana | *S. melongena* | James Robson | London Heathrow AP | 04-IX-2011 | 21116401 | 610 |
| *L. africensis* | F | Ghana | *Solanum sp.* | Ged Hayward | London Heathrow AP | 16-VI-2011 | 21111254 | 607 |
| *L. africensis* | F | Ghana | *S. melongena* | Chongboi Haokip | London Heathrow AP | 13-X-2010 | 21023428 | 617 |
| *L. africensis* | M | ?Ghana | *S. melongena* | Angela Donoghue | Manchester AP | 10-VII-2011 | 21112763A | 600 |
| *L. africensis* | 1 pu | ?Ghana | *S. melongena* | Angela Donoghue | Manchester AP | 10-VII-2011 | 21112763B | - |
| *L. laisalis* | ? | Ghana | *S. melongena* | Keith Warwick | Manchester AP | 15-VI-2009 | 20906676A | - |
| *L. laisalis* | ? | Ghana | *S. melongena* | Keith Warwick | Manchester AP | 15-VI-2009 | 20906676B | - |
| *L. laisalis* | ? | Ghana | *S. melongena* | unknown | London Heathrow AP | 07-XI-2006 | 20621095A | 603 |
| *L. laisalis* | ? | Ghana | *S. melongena* | unknown | London Heathrow AP | 07-XI-2006 | 20621095B | 617 |
| *L. laisalis* | ? | Ghana | *S. melongena* | James Robson | London Heathrow AP | 06-IX-2011 | 21116401 | 617 |
| *L. laisalis* | ? | Ghana | *S. melongena* | Nicholas Finnis | London Heathrow AP | 13-VIII-2012 | 21214373A | 603 |
| *L. laisalis* | ? | Ghana | *S. melongena* | Nicholas Finnis | London Heathrow AP | 04-VII-2011 | 21112293B | 617 |
| *L. laisalis* | ? | Ghana | *S. melongena* | Nicholas Finnis | London Heathrow AP | 04-VII-2011 | 21112293C | 617 |
| *L. laisalis* | ? | Ghana | *S. melongena* | Nicholas Finnis | London Heathrow AP | 04-VII-2011 | 21112293D | 603 |
| *L. laisalis* | ? | Kenya | *S. melongena* | Keith Warwick | Manchester AP | 21-III-2010 | 21005491A | - |
| *L. laisalis* | ? | Kenya | *S. melongena* | Keith Warwick | Manchester AP | 21-III-2010 | 21005491B | - |
| *L. laisalis* | ? | Kenya | *S. melongena* | Keith Warwick | Manchester AP | 21-III-2010 | 21005491C | - |
| *L. laisalis* | ? | Kenya | *S. melongena* | James Robson | London Heathrow AP | 29-V-2012 | 21209240A | 617 |
| *L. laisalis* | ? | Kenya | *S. melongena* | James Robson | London Heathrow AP | 29-V-2012 | 21209240B | 618 |
| *L. laisalis* | ? | Kenya | *S. melongena* | James Robson | London Heathrow AP | 29-V-2012 | 21209240C | 617 |
| *L. laisalis* | 1 lv | Kenya | *S. melongena* | James Robson | London Heathrow AP | 29-V-2012 | 21209240D | - |
| *L. orbonalis* | M | Bangladesh | *S. melongena* | Maureen Tierney | London Heathrow AP | 20-X-2008 | 20818493A | - |
| *L. orbonalis* | F | Bangladesh | *S. melongena* | Maureen Tierney | London Heathrow AP | 20-X-2008 | 20818493B | 613 |
| *L. orbonalis* | M | Bangladesh | *S. melongena* | Simon Honey | London Heathrow AP | 29-IX-2011 | 21118821A | 618 |
| *L. orbonalis* | F | Bangladesh | *S. melongena* | Simon Honey | London Heathrow AP | 29-IX-2011 | 21118821B | 607 |
| *L. orbonalis* | F | Bangladesh | *S. melongena* | Simon Honey | London Heathrow AP | 29-IX-2011 | 21118821C | 616 |
| *L. orbonalis* | M | Bangladesh | *S. melongena* | Sameer Bandali | London Heathrow AP | 03-V-2010 | 21008744A | 616 |
| *L. orbonalis* | F | Bangladesh | *S. melongena* | Sameer Bandali | London Heathrow AP | 03-V-2010 | 21008744B | 616 |
| *L. orbonalis* | F | Bangladesh | *S. melongena* | Ian Hartley | London Heathrow AP | 04-IX-2010 | 21019143 | 609 |
| *L. orbonalis* | M | India | *S. melongena* | Commercial sample | Commercial sample | 15-VI-2011 | 21111285A | 619 |
| *L. orbonalis* | M | India | *S. melongena* | Commercial sample | Commercial sample | 15-VI-2011 | 21111285B | 607 |
| *L. orbonalis* | F | India | *S. melongena* | Commercial sample | Commercial sample | 15-VI-2011 | 21111285C | 607 |
| *L. orbonalis* | M | Laos | *S. melongena* | Joanna Wyzkiewicz | London Heathrow AP | 18-IX-2010 | 21020895A | - |
| *L. orbonalis* | M | Laos | *S. melongena* | Joanna Wyzkiewicz | London Heathrow AP | 18-IX-2010 | 21020895B | - |
| *L. orbonalis* | M | Laos | *S. melongena* | Joanna Wyzkiewicz | London Heathrow AP | 18-IX-2010 | 21020895C | 600 |
| *L. orbonalis* | F | Laos | *S. melongena* | Joanna Wyzkiewicz | London Heathrow AP | 18-IX-2010 | 21020895D | 617 |
| *L. orbonalis* | F | Laos | *S. melongena* | Joanna Wyzkiewicz | London Heathrow AP | 18-IX-2010 | 21020894A | 609 |
| *L. orbonalis* | F | Laos | *S. melongena* | Joanna Wyzkiewicz | London Heathrow AP | 18-IX-2010 | 21020894B | 610 |
| *L. orbonalis* | M | Malaysia | *S. melongena* | Angela Donoghue | Manchester AP | 17-VII-2011 | 21113260 | 618 |
| *L. orbonalis* | F | Pakistan | *S. melongena* | Angela Donoghue | Manchester AP | 16-XI-2011 | 21122619A | - |
| *L. orbonalis* | F | Pakistan | *S. melongena* | Angela Donoghue | Manchester AP | 16-XI-2011 | 21122619B | 619 |
| *L. orbonalis* | M | Pakistan | *S. melongena* | Angela Donoghue | Manchester AP | 12-X-2011 | 21120109A | 606 |
| *L. orbonalis* | 1 lv | Pakistan | *S. melongena* | Angela Donoghue | Manchester AP | 12-X-2011 | 21120109B | - |
| *L. orbonalis* | M | Pakistan | *S. melongena* | Angela Donoghue | Manchester AP | 18-IX-2011 | 21117738 | 618 |
| *L. orbonalis* | M | Pakistan | *S. melongena* | Angela Donoghue | Manchester AP | 02-X-2011 | 21119034A | 600 |
| *L. orbonalis* | F | Pakistan | *S. melongena* | Angela Donoghue | Manchester AP | 02-X-2011 | 21119034B | 606 |
| *L. orbonalis* | M | Pakistan | *S. melongena* | Caroline Cawood | Manchester AP | 12-III-2009 | 20904861 | 617 |
| *L. orbonalis* | 5 lv | Pakistan | *S. melongena* | Angela Donoghue | Manchester AP | 02-X-2011 | 21119034A | - |
| *L. orbonalis* | 1 pu | Pakistan | *S. melongena* | Angela Donoghue | Manchester AP | 02-X-2011 | 21119034B | - |
| *L. orbonalis* | F | Pakistan | *S. melongena* | Martin Parry | Manchester AP | 22-VIII-2011 | 21115591 | 597 |
| *L. orbonalis* | F | Pakistan | *S. melongena* | Michael Gibbs | London Heathrow AP | 30-III-2011 | 21106042 | 607 |
| *L. orbonalis* | 1 lv | Pakistan | *S. Melongena* | Ian Hartley | London Heathrow AP | 12-IX-2011 | 21117039 | - |
| *L. orbonalis* | F | Sri Lanka | *S. melongena* | Michael Gibbs | London Heathrow AP | 08-IV-2011 | 21106809 | 610 |
| *L. orbonalis* | F | Sri Lanka | *S. xanthocarpum* | Nicholas Finnis | London Heathrow AP | 05-IV-2012 | 21205998 | 617 |
| *L. orbonalis* | F | Sri Lanka | *S. xanthocarpum* | Nicholas Finnis | London Heathrow AP | 12-II-2012 | 21202345 | 582 |
| *L. orbonalis* | M | Thailand | *S. melongena* | Caroline Cawood | Manchester AP | 29-VI-2009 | 20911505 | 583 |
| *L. orbonalis* | F | Thailand | *S. melongena* | unkown | London Heathrow AP | 07-VIII-2008 | 20812939 | 617 |
| *L. orbonalis* | M | unknown | *S. melongena* | Commercial sample | Commercial sample | 07-XI-2011 | 21122121A | 618 |
| *L. orbonalis* | M | unknown | *S. melongena* | Commercial sample | Commercial sample | 07-XI-2011 | 21122121B | 617 |
| *L. orbonalis* | M | unknown | *S. melongena* | Commercial sample | Commercial sample | 07-XI-2011 | 21122121C | - |
| *L. orbonalis* | M | unknown | *S. melongena* | Commercial sample | Commercial sample | 07-XI-2011 | 21122121D | 618 |
| *L. orbonalis* | 1 lv | unknown | *S. melongena* | Commercial sample | Commercial sample | 07-XI-2011 | 21122121E | - |
| *L. pseudorbonalis* | F | Uganda | *S. aethiopicum* | Commercial sample | Commercial sample | 23-IV-2013 | 21307410 | 617 |
| *N. elegantalis* | F | Colombia | *Cyphomandra betacea* | Ged Hayward | London Heathrow AP | 11-VI-2013 | 21309643A | 618 |
| *N. elegantalis* | F | Colombia | *Cyphomandra betacea* | Ged Hayward | London Heathrow AP | 11-VI-2013 | 21309643B | 617 |
