# Supplementary material for: Discovery of an unknown diversity of Leucinodes species damaging Solanaceae fruits in sub-Saharan Africa and moving in trade (Insecta, Lepidoptera, Pyraloidea)
Source: Zookeys. 2015 Jan 19;(472):117–62. doi: 10.3897/zookeys.472.8781 (PMC4304033; doi:10.3897/zookeys.472.8781)
Supplement: Supplementary material 2 — Overview of barcoded specimens. [file zookeys-472-117-s002.docx]

# Appendix II

**Overview of barcoded specimens.**

Abbreviations: bp - base pairs; ENA - European Nucleotide Archive.

^1^ Chakrabarty et al. (2013) GenSeq: An updated nomenclature and ranking for genetic sequences from type and non-type sources. ZooKeys 346: 29-41. doi: 10.3897/zookeys.346.5753

| **Species** | **Origin** | **voucher number** | **ENA/GenBank accession number** | **Barcode length [bp]** | **GenSeq Nomenclature^1^** |
| --- | --- | --- | --- | --- | --- |
| *Leucinodes africensis* | Angola | SMTD Lep1562 | LN624711 | 658 | genseq-3 |
| *Leucinodes africensis* | Angola | SMTD Lep1563 | LN624712 | 640 | genseq-3 |
| *Leucinodes africensis* | Ghana | Fera21026690 | LN624713 | 601 | genseq-3 |
| *Leucinodes africensis* | Ghana | Fera21027623A | LN624714 | 598 | genseq-3 |
| *Leucinodes africensis* | Ghana | Fera21115509A | LN624715 | 618 | genseq-3 |
| *Leucinodes africensis* | Ghana | Fera21115509B | LN624716 | 617 | genseq-3 |
| *Leucinodes africensis* | Ghana | Fera21115509C | LN624717 | 617 | genseq-3 |
| *Leucinodes africensis* | Ghana | Fera21115499 | LN624718 | 617 | genseq-3 |
| *Leucinodes africensis* | Ghana | Fera21104230A | LN624719 | 617 | genseq-3 |
| *Leucinodes africensis* | Ghana | Fera21104230B | LN624720 | 616 | genseq-3 |
| *Leucinodes africensis* | Ghana | Fera21104230C | LN624737 | 617 | genseq-3 |
| *Leucinodes africensis* | Ghana | Fera21027623B | LN624721 | 618 | genseq-3 |
| *Leucinodes africensis* | Ghana | Fera21111730 | LN624722 | 601 | genseq-3 |
| *Leucinodes africensis* | Ghana | Fera21103712 | LN624724 | 609 | genseq-3 |
| *Leucinodes africensis* | Ghana | Fera21114408 | LN624725 | 610 | genseq-3 |
| *Leucinodes africensis* | Ghana | Fera21115509D | LN624726 | 610 | genseq-3 |
| *Leucinodes africensis* | Ghana | Fera21115509E | LN624727 | 611 | genseq-3 |
| *Leucinodes africensis* | Ghana | Fera21115509F | LN624728 | 610 | genseq-3 |
| *Leucinodes africensis* | Ghana | Fera21116401A | LN624729 | 610 | genseq-3 |
| *Leucinodes africensis* | Ghana | Fera21111170 | LN624730 | 610 | genseq-3 |
| *Leucinodes africensis* | Ghana | Fera21027623D | LN624731 | 610 | genseq-3 |
| *Leucinodes africensis* | Ghana | Fera21027623C | LN624732 | 616 | genseq-3 |
| *Leucinodes africensis* | Ghana | Fera21111254 | LN624733 | 607 | genseq-3 |
| *Leucinodes africensis* | Ghana | Fera21027623E | LN624734 | 605 | genseq-3 |
| *Leucinodes africensis* | Ghana | Fera21023428 | LN624735 | 617 | genseq-3 |
| *Leucinodes africensis* | Ghana | Fera21027623F | LN624736 | 601 | genseq-3 |
| *Leucinodes africensis* | Ghana | SMTD Lep946 | LN624710 | 655 | genseq-3 |
| *Leucinodes africensis* | ?Ghana | Fera21112763 | LN624723 | 600 | genseq-3 |
| *Leucinodes kenyensis* | Kenya | USNM ENT 00719337 | HQ947382 | 654 | genseq-1 |
| *Leucinodes kenyensis* | Kenya | USNM ENT 00719338 | HQ947383 | 654 | genseq-2 |
| *Leucinodes kenyensis* | Kenya | USNM ENT 00719339 | HQ947384 | 654 | genseq-2 |
| *Leucinodes kenyensis* | Kenya | USNM ENT 00719976 | KM987390 | 654 | genseq-2 |
| *Leucinodes africensis* | Nigeria | USNM ENT 00196725 | KM987391 | 654 | genseq-3 |
| *Leucinodes laisalis* | Ghana | Fera20621095A | LN624738 | 603 | genseq-3 |
| *Leucinodes laisalis* | Ghana | Fera20621095B | LN624739 | 617 | genseq-3 |
| *Leucinodes laisalis* | Kenya | Fera21209240A | LN624740 | 617 | genseq-3 |
| *Leucinodes laisalis* | Kenya | Fera21209240B | LN624741 | 618 | genseq-3 |
| *Leucinodes laisalis* | Kenya | Fera21209240C | LN624742 | 617 | genseq-3 |
| *Leucinodes laisalis* | Ghana | Fera21116401B | LN624743 | 617 | genseq-3 |
| *Leucinodes laisalis* | Ghana | Fera21214373 | LN624744 | 603 | genseq-3 |
| *Leucinodes laisalis* | Ghana | Fera21112293A | LN624745 | 617 | genseq-3 |
| *Leucinodes laisalis* | Ghana | Fera21112293B | LN624746 | 617 | genseq-3 |
| *Leucinodes laisalis* | Ghana | Fera21112293C | LN624747 | 603 | genseq-3 |
| *Leucinodes laisalis* | Kenya | USNM 196701 | KM987393 | 618 | genseq-3 |
| *Leucinodes laisalis* | Kenya | USNM ENT 00196697 | KM987396 | 654 | genseq-3 |
| *Leucinodes laisalis* | Kenya | USNM ENT 00196699 | KM987395 | 654 | genseq-3 |
| *Leucinodes laisalis* | Kenya | USNM ENT 00196700 | KM987394 | 654 | genseq-3 |
| *Leucinodes laisalis* | Kenya | USNM ENT 00196702 | KM987403 | 654 | genseq-3 |
| *Leucinodes laisalis* | Kenya | USNM ENT 00196703 | KM987402 | 654 | genseq-3 |
| *Leucinodes laisalis* | Kenya | USNM ENT 00196704 | KM987401 | 612 | genseq-3 |
| *Leucinodes laisalis* | Kenya | USNM ENT 00196705 | KM987400 | 654 | genseq-3 |
| *Leucinodes laisalis* | Kenya | USNM ENT 00196706 | KM987399 | 654 | genseq-3 |
| *Leucinodes laisalis* | Nigeria | USNM ENT 00676643 | KM987397 | 654 | genseq-3 |
| *Leucinodes laisalis* | Kenya | USNM ENT 00719748 | KM987398 | 654 | genseq-3 |
| *Leucinodes laisalis* | South Africa | BC MTD 01819 | KM987697 | 657 | genseq-3 |
| *Leucinodes malawiensis* | Malawi | SMTD Lep1617 | LN624676 | 658 | genseq-1 |
| *Leucinodes orbonalis* | Pakistan | Fera21122619B | LN624679 | 619 | genseq-3 |
| *Leucinodes orbonalis* | Unknown | Fera21122121A | LN624680 | 618 | genseq-3 |
| *Leucinodes orbonalis* | Unknown | Fera21122121B | LN624681 | 617 | genseq-3 |
| *Leucinodes orbonalis* | Unknown | Fera21122121D | LN624682 | 618 | genseq-3 |
| *Leucinodes orbonalis* | Pakistan | Fera21120109 | LN624683 | 606 | genseq-3 |
| *Leucinodes orbonalis* | Bangladesh | Fera21008744A | LN624684 | 616 | genseq-3 |
| *Leucinodes orbonalis* | Pakistan | Fera21117738 | LN624685 | 618 | genseq-3 |
| *Leucinodes orbonalis* | Bangladesh | Fera21118821A | LN624686 | 618 | genseq-3 |
| *Leucinodes orbonalis* | Pakistan | Fera21119034A | LN624687 | 600 | genseq-3 |
| *Leucinodes orbonalis* | Laos | Fera21020895C | LN624688 | 600 | genseq-3 |
| *Leucinodes orbonalis* | Malaysia | Fera21113260 | LN624689 | 618 | genseq-3 |
| *Leucinodes orbonalis* | India | Fera21111285A | LN624690 | 619 | genseq-3 |
| *Leucinodes orbonalis* | Pakistan | Fera20904861 | LN624691 | 617 | genseq-3 |
| *Leucinodes orbonalis* | India | Fera21111285B | LN624692 | 607 | genseq-3 |
| *Leucinodes orbonalis* | Bangladesh | Fera21118821B | LN624693 | 607 | genseq-3 |
| *Leucinodes orbonalis* | Bangladesh | Fera21118821C | LN624694 | 616 | genseq-3 |
| *Leucinodes orbonalis* | Pakistan | Fera21119034B | LN624695 | 606 | genseq-3 |
| *Leucinodes orbonalis* | Pakistan | Fera21115591 | LN624696 | 597 | genseq-3 |
| *Leucinodes orbonalis* | Pakistan | Fera21106042 | LN624697 | 607 | genseq-3 |
| *Leucinodes orbonalis* | Bangladesh | Fera21008744B | LN624698 | 616 | genseq-3 |
| *Leucinodes orbonalis* | India | Fera21111285C | LN624699 | 607 | genseq-3 |
| *Leucinodes orbonalis* | Bangladesh | Fera20818493B | LN624700 | 613 | genseq-3 |
| *Leucinodes orbonalis* | Bangladesh | Fera21019143 | LN624701 | 609 | genseq-3 |
| *Leucinodes orbonalis* | Laos | Fera21020894A | LN624702 | 609 | genseq-3 |
| *Leucinodes orbonalis* | Laos | Fera21020895D | LN624703 | 617 | genseq-3 |
| *Leucinodes orbonalis* | Sri Lanka | Fera21106809 | LN624704 | 610 | genseq-3 |
| *Leucinodes orbonalis* | Laos | Fera21020894B | LN624705 | 610 | genseq-3 |
| *Leucinodes orbonalis* | Thailand | Fera20911505 | LN624706 | 583 | genseq-3 |
| *Leucinodes orbonalis* | Thailand | Fera20812939 | LN624707 | 617 | genseq-3 |
| *Leucinodes orbonalis* | Sri Lanka | Fera21205998 | LN624708 | 617 | genseq-3 |
| *Leucinodes orbonalis* | Sri Lanka | Fera21202345 | LN624709 | 582 | genseq-3 |
| *Leucinodes pseudorbonalis* | Uganda | Fera21307410 | LN624674 | 617 | genseq-3 |
| *Leucinodes rimavallis* | Kenya | SMTD Lep1592 | LN624677 | 589 | genseq-3 |
| *Leucinodes rimavallis* | Kenya | SMTD Lep1593 | LN624678 | 658 | genseq-3 |
| *Leucinodes* sp. | Namibia | SMTD Lep1872 | LN624675 | 615 | - |
| *Leucinodes* sp. | Swaziland | BC MTD 01818 | KM987698 | 633 | - |
| *Neoleucinodes dissolvens* | French Guiana | SMTD Lep1046 | LN624670 | 655 | genseq-4 |
| *Neoleucinodes elegantalis* | Colombia | Fera21309643A | LN624672 | 618 | genseq-4 |
| *Neoleucinodes elegantalis* | Colombia | Fera21309643B | LN624673 | 617 | genseq-4 |
| *Neoleucinodes elegantalis* | French Guiana | SMTD Lep780 | LN624668 | 627 | genseq-4 |
| *Neoleucinodes* sp. 1 | Dominican Republic | SMTD Lep947 | LN624669 | 654 | genseq-4 |
| *Neoleucinodes* sp. 1 | Brazil | SMTD Lep1124 | LN624671 | 658 | genseq-4 |
| *Neoleucinodes* sp. 2 | Costa Rica | BC MTD 01186 | JN284843 | 654 | genseq-4 |
| *Analyta cf. apicalis* | Papua New Guinea | USNM ENT 00732968 | KM987389 | 654 | genseq-4 |
| *Lygropia aureomarginalis* | Sierra Leone | USNM ENT 00732974 | KM987392 | 615 | genseq-4 |
| *Lygropia* cf. *aureomarginalis* | Tanzania | SMTD Lep1618 | LN624666 | 658 | genseq-4 |
| *Lygropia* sp. | Papua New Guinea | SMTD Lep1596 | LN624665 | 654 | genseq-4 |
| *Syllepte vagans* | Nigeria | USNM ENT 00196724 | KM987404 | 654 | genseq-4 |
| *Syllepte* cf. *vagans* | Uganda | SMTD Lep1620 | LN624667 | 615 | genseq-4 |
| *Udea ferrugalis* | Morocco | SMTD Lep870 | LN624664 | 657 | genseq-4 |
